# Models of integrated care for older people with frailty: a horizon scanning review

**DOI:** 10.1136/bmjopen-2021-060142

**Published:** 2022-04-08

**Authors:** Ashwanee A Kjelsnes, Eli Feiring

**Affiliations:** Department of Health Management and Health Economics, University of Oslo, Oslo, Norway

**Keywords:** health policy, organisation of health services, health services administration & management

## Abstract

**Objectives:**

Frailty, a multifaceted geriatric condition, is an emerging global health problem. Integrated care models designed to meet the complex needs of the older people with frailty are required. Early identification of innovative models may inform policymakers and other stakeholders of service delivery alternatives they can introduce and locally adapt so as to tackle system fragmentation and lack of coordination. This study used horizon scanning methodologies to systematically search for, prioritise and assess new integrated care models for older people with frailty and investigated experts’ views on barriers and facilitators to the adoption of horizon scanning in health services research.

**Methods:**

A four-step horizon scanning review was performed. Frailty-specific integrated care models and interventions were identified through a review of published literature supplemented with grey literature searches. Results were filtered and prioritised according to preset criteria. An expert panel focus group session assessed the prioritised models and interventions on innovativeness, impact and potential for implementation. The experts further evaluated horizon scanning for its perceived fruitfulness in aiding decision-making.

**Results:**

Nine integrated care models and interventions at system level (n=5) and community level (n=4) were summarised and assessed by the expert panel (n=7). Test scores were highest for the Walcheren integrated care model (system-based model) and EuFrailSafe (community-based intervention). The participants stated that horizon scanning as a decision-making tool could aid in assessing knowledge gaps, criticising the status quo and developing new insights. Barriers to adoption of horizon scanning on individual, organisational and wider institutional level were also identified.

**Conclusion:**

Study findings demonstrated that horizon scanning is a potentially valuable tool in the search for innovative service delivery models. Further studies should evaluate how horizon scanning can be institutionalised and effectively used for serving this purpose.

**Strengths and limitations of this study**

- The unique contribution of this study is its use of horizon scanning methodologies to identify promising integrated care models and interventions.
- The study’s main strength is its systematic method of information mapping, filtration, prioritisation and assessment.
- A limitation is that service models are often already established as practices when reported, thus it is difficult to scan for new interventions in this context.
- A further limitation is that the transferability of results to other setting may be limited.

## Introduction

Frailty, a multifaceted geriatric condition characterised by increased vulnerability to stress incidents due to reductions in reserve and functions in multiple physiological systems, is emerging as a global health problem with significant clinical and public health consequences.^1–4^ It is approximated that 21.3% of the world’s population will be 60 years or older by 2050, where frailty is estimated to affect around one out of every six community-dwelling seniors.^5^ Frailty is associated with a significant increase in comorbid chronic illnesses, functional dependency, disability, healthcare needs and costs.^6 7^ To avoid or delay the progression of frailty to significant functional decline, healthcare designed to meet the complex care requirements is needed.^1 8–11^ In Norway, as in many other countries, establishing high-quality integrated care for older people with frailty is a political priority.^12^ Integrated care, understood as comprehensive, multilevel and across settings of organisation of care, is generally believed to be a solution to the demand for improved care for the multimorbid and long-term care patients.^13^ However, a recent systematic review on integrated care models for managing and preventing frailty concluded that few models were specifically designed to prevent and tackle frailty in the community and at the interface between primary care and secondary care.^14^

The absence of a standardised frailty definition and assessment method coupled with the fact that literature on frailty-specific integrated care models and interventions is still in its early stages of development makes it challenging for healthcare decision-makers to meet the needs of the older people with frailty.^15–17^ The search for signals of important development in this context can possibly be lessened by horizon scanning, which acts as an information resource that can aid in decisions about the identification of innovative healthcare interventions.^18^

Horizon scanning is a systematic approach for detecting early signals of potentially important developments that could impact areas of interest.^19^ It involves a comprehensive review of data to bridge knowledge gaps, question assumptions, assess possible threats, challenges and emerging problems, as well as look for opportunities to present new policy alternatives.^20–23^ Signals of ‘things to come’ are detected from manifold information sources in addition to, or even instead of, reviews of scientific literature. These sources include targeted literature searches and input from expert groups, committees, surveys, government bodies, conferences, associations, media and more. Further, experts and other stakeholders with diverse views, experiences and roles may be brought together to systematically discuss signals as part of the horizon scanning process.

In healthcare, horizon scanning methodologies are commonly used as a health technology assessment tool in early awareness and alert systems (EAAS) of pharmaceuticals to allow for innovative medicines to enter the market. Less attention has been given to the employment of horizon scanning methodologies in identifying improvements for delivery of healthcare services.^24^

At this backdrop, we wanted to investigate if employing horizon scanning methodologies could be a valuable and viable strategy to identify novel integrated care initiatives for older people with frailty, in an early phase of adoption. First, we aimed to identify new and emerging integrated care models and interventions that could potentially address system fragmentation issues faced by the older people with frailty and use the opinions of experts to evaluate these models and interventions based on their level of innovation, probability of implementation and impact.

The second aim was to look into experts’ opinions on the fruitfulness of employing horizon scanning methodologies in this context, given horizon scanning is still a relatively new tool for identifying innovative healthcare delivery models.

## Methods

### Study design

This study was designed as a small-scale horizon scanning. The Preferred Reporting Items for Systematic Reviews and Meta-Analyses guidelines were used to report the literature search process as far as possible, and the Consolidated criteria for Reporting Qualitative research guidelines were used to report the findings from the qualitative focus group (online supplemental files 1 and 2).

10.1136/bmjopen-2021-060142.supp1 Supplementary data

10.1136/bmjopen-2021-060142.supp2 Supplementary data

### Setting

The Norwegian healthcare system is universal, tax financed and semidecentralised.^25^ The responsibility for primary health and social care lies with the municipalities. The central state is responsible for secondary and specialist healthcare, which is administrated by four Regional Health Authorities. The lack of communication between the two tiers of governance contributes to challenges with delivery of integrated care.^26^ Although a Coordination Reform (2012) established mandatory network governance to improve coordination between primary and specialist care, integrated care involving different levels is hindered by lack of formalised coordination and cooperation between the municipalities and the hospitals.^12^

### Horizon scanning to identify novel integrated care models

**Figure 1:**
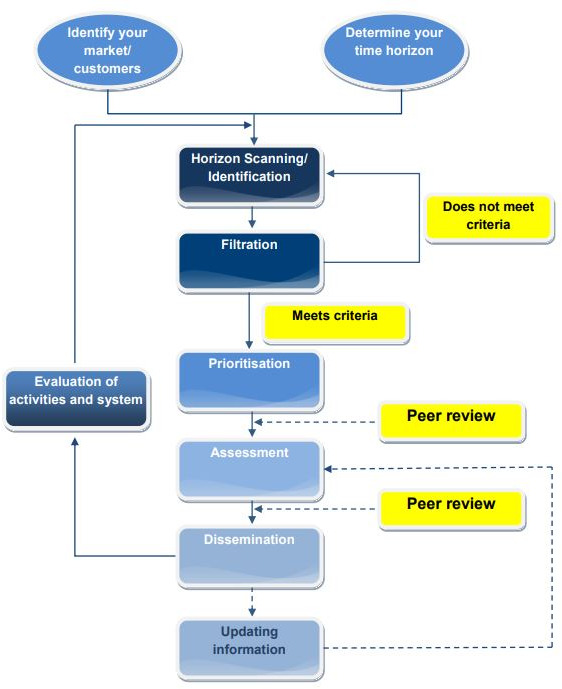
Common stages of horizon scanning from the EuroScan Network.^24^ This figure is licensed under the Creative Commons Attribution-NonCommercial-ShareAlike 4.0 International license.

Horizon scanning generally follows a six-step approach of signal detection, filtration, prioritisation, assessment, and dissemination and updating information (figure 1). The first step often includes mapping signals of innovation with the use of literature reviews, including reviews of grey literature and reports retrieved from governmental bodies, conferences, meetings, forums, observatories and other organisations. Preset filtration and prioritisation criteria are used to discard irrelevant signals. Assessment methods include participation of experts, users and policymakers, and peer reviews. The results of the horizon scanning are then disseminated and evaluated.^24^

A horizon scanning may be carried out at the beginning of a broader foresight process, aiming to address the full cycle of policy on ‘complex futures’ and involving a range of stakeholders, long-term considerations and different scenarios. It may, however, also be a standalone approach for identifying ‘things to come’. In the present study, the horizon scanning process carried out followed the first four steps of the EuroScan methods toolkit for EAAS.^24^ We conducted a focus group session to obtain thoughts on integrated care needs for older people with frailty, as well as opinions on the models and interventions identified in the literature and perspectives on horizon scanning methodologies and their potential consequences.

**Figure 2:**
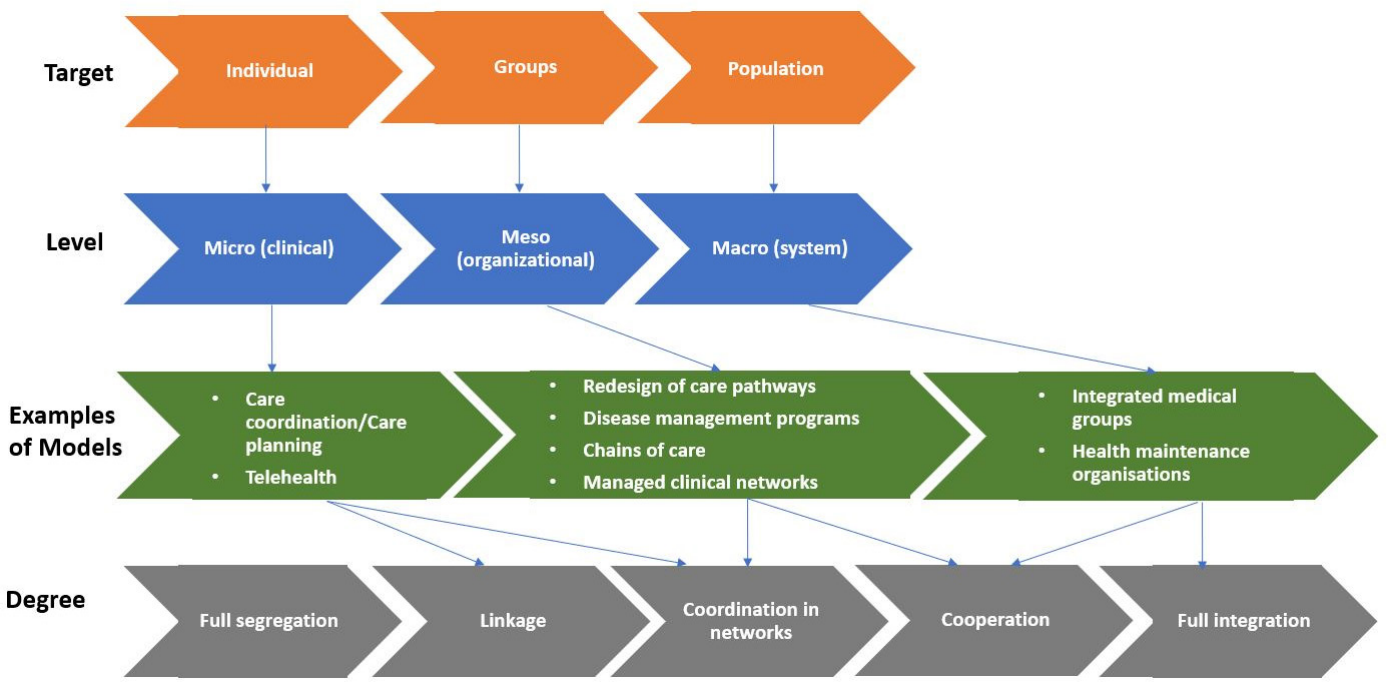
Integrated care models. Adapted from 27–29.

We followed a multifaceted definition of ‘integrated care’ in this study. Integrated care models can be organised according to target group, level and degree (figure 2). Thus, we kept a broad understanding of integrated care as an organisational coordination mechanism that can be understood as to providing a cohesive and continuum of care that is personalised to the patient’s condition.^27–29^

### Literature search strategy: identification, filtration and prioritisation of records

#### Search strategy

Reviews of published literature and grey literature were performed to trace new and emerging integrated care models and interventions, targeted at the older people with frailty, which had the potential for addressing system fragmentation issues. Databases and governmental bodies were searched using prespecified search terms to identify research papers, proceedings of conferences and workshops, policy papers and reports (table 1). Only records published in English or Norwegian were included. The final search took place from 11 January 2020 to 02 January 2021.

**Table 1:** Information sources and search terms used for signal detection

Information sources	Search terms
Online databases PubMed (384) Cochrane Library (19) Evidence-based medical reviews (24) Embase (349) Oria UiO (50) JStor (92) Medline Ovid (27) Web of Science (41) Scopus (104)	Frail elderly Integrated care model Multidisciplinary Aged care Service delivery model Older people Geriatric >65 Health sciences Political sciences Public health Public policy and administration Health policy
Governmental reports and conferences Norwegian Institute of Public Health (20) The Norwegian Directorate of Health (29) Ministry of Health and Care Services (10) Norwegian National Advisory Unit on Ageing and Health (28) The innovation conference: the outward-looking hospital (1) Frailty among the elderly conference (1)	

#### Inclusion and exclusion criteria

Findings were filtered by scanning each record’s abstract, title and keywords based on a set of inclusion and exclusion criteria, which were adapted from EuroScan^24^ and the National Horizon Scanning Centre (NHSC) guidelines for horizon scanning,^30^ as well as from previous literature. Records that dealt with the adoption, execution or assessment of initiatives focused on the concept of patient-centred integration: funding, administrative, organisational, service delivery, and clinical levels required to promote interaction, coordination, and cooperation in and between the cure and care sectors were included.^15^

Records focused solely on integrated care, multidisciplinary team and frailty without describing any intervention and/or model, as well as those not specifically focused on the older people with frailty, were excluded. Disease-specific publications were removed because frailty is considered a multifaceted and dynamic disease.^31–38^

A range of integrated care models and interventions were identified in the material. The different initiatives have been developed to be applied at different key points in the frailty care pathway,^39^ such as preventive education, enablement and care and support at home, assessment at management in primary care, geriatric assessment in hospital and intermediate care services.^39^ We chose to group the remaining records into two groups with an aim to create a better overview for discussion and evaluation. First, we identified initiatives developed to give comprehensive integrated chronic care and we categorised these models as ‘system-level integrated care models’. Second, we categorised more discrete interventions that provide specific components of integrated care as ‘community-based interventions’. The grouping was not unambiguous as the integrated care models and community-based interventions do contain overlapping elements. We included records that described models that had some or all of the characteristics illustrated in table 2.

**Table 2:** Intervention characteristics and considerations used to filter and prioritise models and interventions^34 59 60^

System-level integrated care models for older people with frailty	Community-based interventions for older people with frailty	Prioritisation criteria
Centralised point of entry Geriatric evaluations Case management Multidisciplinary teams Multidisciplinary guidelines and meetings Digitalised patient files Network framework	Local or community level-based interventions Living-at-home Measures described to promote independence	Potential care outcomes Potential cost-effectiveness Expected resource utilisation Expected reorganisation of services Applicability Novelty Forward thinking

#### Prioritisation of models and interventions prior to focus group assessment

Prior to focus group assessment, we did a criteria-informed qualitative prioritisation of the system-based models and community-based interventions (table 2). The aim of the prioritisation was to identify models and interventions not yet implemented or tested in a Norwegian setting, which we considered to have the potential to address system fragmentation issues.

### Focus group: assessment of records

#### Participants and recruitment

The focus group’s goal was to discuss and assess the literature review’s findings. Purposive sampling was used to recruit participants who had a variety of roles and educational backgrounds as well as knowledge of services provided to the older people with frailty.^40^ The research team approached the Norwegian National Advisory Unit on Ageing and Health and was set in contact with potential participants who were subsequently invited to the study. The invitees further provided potential participants (snowball sampling). Eleven persons were invited to participate.

#### Data collection

The focus group was conducted on 07 April 2021 via Zoom by AAK. Consent forms were signed and collected prior to the focus group.

Prior to the focus group discussion, the participants were emailed information on the horizon scanning process conducted, tables of the identified models and interventions, as well as the semistructured topic guide (online supplemental figure 1). They were asked to score and evaluate the different models independently, but we did not collect their evaluations before the focus group took place. This was a pragmatic choice given the study’s time and resource limits.

10.1136/bmjopen-2021-060142.supp3 Supplementary data

The focus group session was divided into three sections. The first section presented a summary of the horizon scanning process as well as the key features of each model and intervention. This was done to clear up any misunderstandings or questions they had about the models and horizon scanning process. The models and interventions were organised and presented in accordance with the various forms of integration, with the aim of demonstrating how they provided complex integrated care to the frail in a clear and understandable manner. To avoid miscommunication among the participants, ‘innovations’ were defined as (1) a possible new way of organising services, (2) a new mechanism in the service process, (3) changes in the system that increase access to more comprehensive services for older people with frailty, (4) a new application of existing intervention(s), or other current innovations.^41^

The second section focused on assessment of the models and interventions where the participants were asked to collectively discuss, reflect and rate each model and intervention on a scale from low to high, on the following equally weighted aspects: level of innovation, probability for implementation in the next 2–10 years and potential impact on the older people with frailty. Further details of what these three aspects meant were also included in the interview guide (online supplemental figure 1). In the third section, participants were finally asked to offer their thoughts on horizon scanning, its prospective implications and potential for use as a decision-making tool.

The focus group session lasted 2 hours. Discussions were recorded on a password-protected computer connected to a university server. The transcription was done through coding to protect the anonymity of the participants.

#### Data analysis

Organisation and analysis of data collected from the focus group discussion followed the continuum of data analysis framework.^42^ Data were transcribed and organised according to the topic guide ensuring that both positive and negative comments with regard to each model and intervention evaluated against the three criteria were included. The descriptive statements were then indexed, arranged, compared, analysed and rearranged to create categories for both quantitative and qualitative results. Data used as illustrative purposes were translated from Norwegian to English by the authors.

#### Patient and public involvement

Patients and public were not involved in any part of our research.

## Results

### Identification, filtration and prioritisation

**Figure 3:**
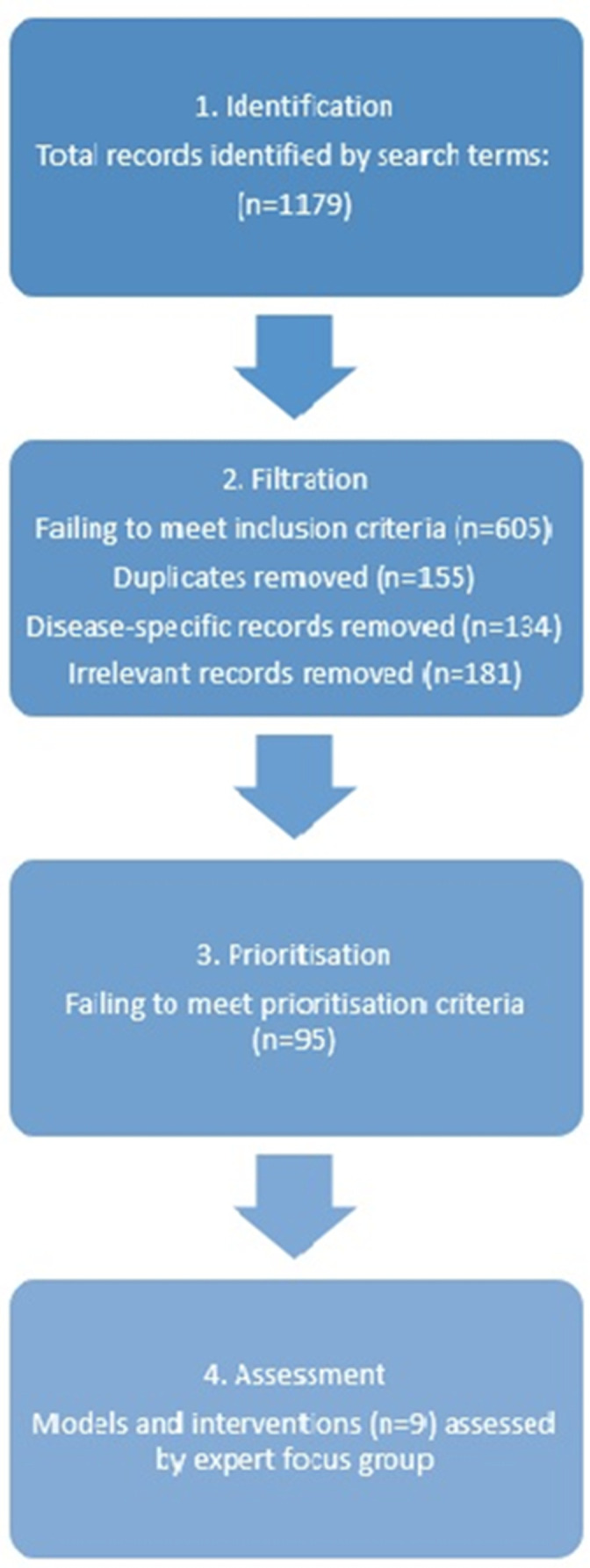
Horizon scanning process chart.

There were 1179 records identified through the initial database searches and grey literature, of which 605 were removed due to failure to meet the inclusion criteria at the filtration stage. One hundred and fifty-five duplicates and 134 disease-specific records were excluded, and 181 records were thereafter removed after reading through the full text for relevance. At the prioritisation stage, 104 records were read and evaluated according to the prioritisation criteria. Nine records were included in this study after prioritisation (figure 3).

**Figure 4:**
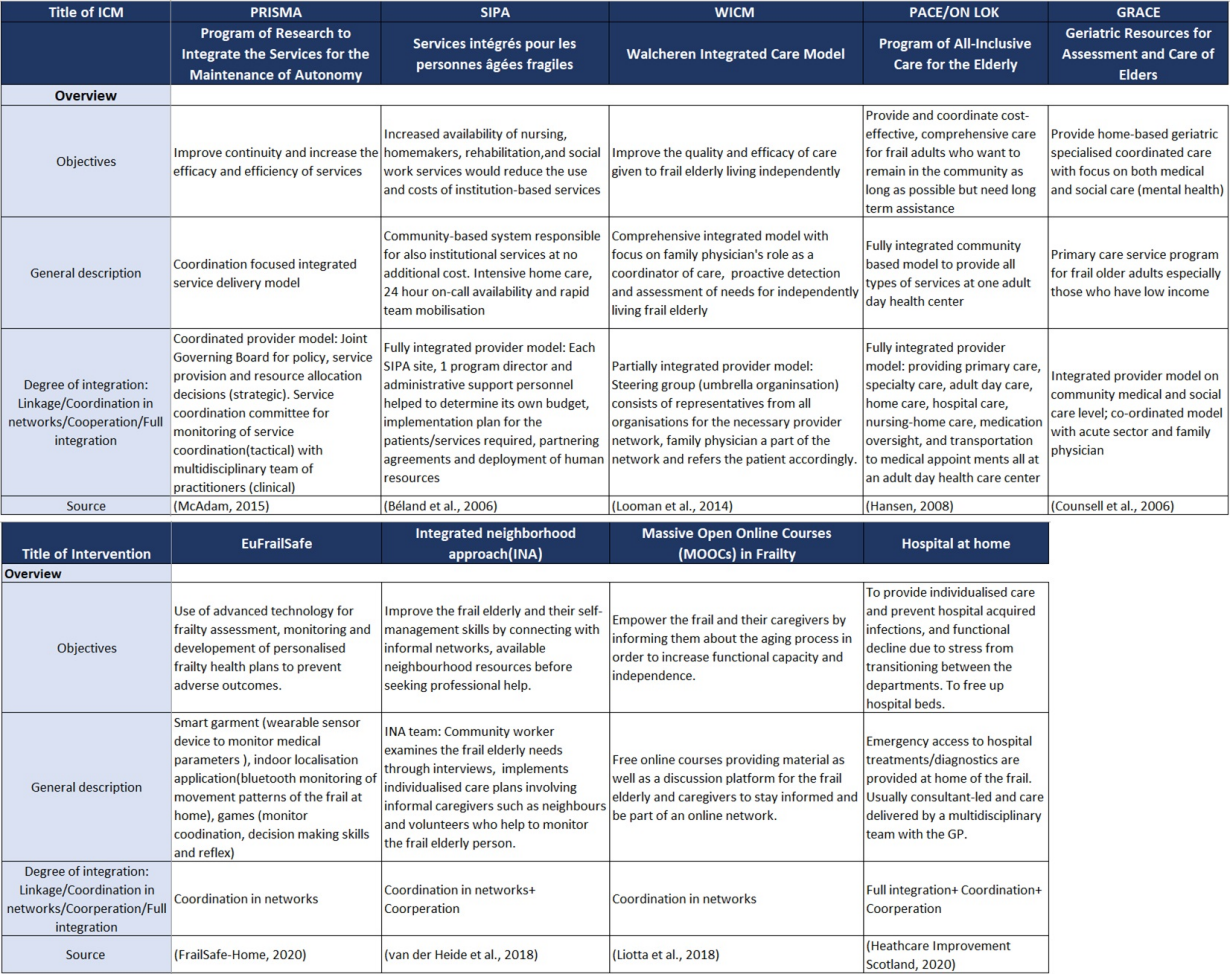
Overview over the models and interventions detailed by the records.^43–51^ GP, general practitioner; ICM, integrated care model.

Five system-based models and four community-based interventions^43–51^ were prioritised to be assessed in the focus group (figure 4), as described in the Methods section.

### Evaluation

#### Participants

Eleven persons were invited to participate in the focus group; four declined the invitation due to other work commitments. The seven participants who took part were experienced healthcare professionals with various educational backgrounds and had multiple roles in academia, specialist and primary care. They resided in different parts of the country (online supplemental table 1).

#### Quantitative scores

The participants discussed and then agreed on a score for each system-level integrated care models and community-based interventions together on the three aspects: innovation, implementation, and impact on a low, moderate, and high scale. The scores are stated below in table 3.

**Table 3:** Scoring of models and interventions

System-based integrated care model	Level of innovation	Probability of implementation in the next 2–10 years	Likely impact on frail elderly
PRISMA	L	L/M	M
SIPA	L	L/M	M
WICM	L/M	H	M/H
PACE	L/M	L	M
GRACE	L/M	L	M
**Community-based intervention**	**Level of innovation**	**Probability of implementation in the next 2–10 years**	**Likely impact on frail elderly**
EuFrailSafe	H	H	H
INA	H	M	M/H
MOOCs	M/H	M/H	M
Hospital at Home	M	M/H	M/H

GRACE, Geriatric Resources for Assessment and Care of Elders; H, high; INA, integrated neighbourhood approach; L, low; M, moderate; MOOCs, massive open online courses; PACE, Program of All-Inclusive Care for the Elderly; PRISMA, Program of Research to Integrate the Services for the Maintenance of Autonomy; SIPA, Services intégrés pour les personnes âgées fragiles; WICM, Walcheren Integrated Care Model.

The Walcheren Integrated Care Model (WICM) had the overall highest scores among the system-based integrated care models. It received low to moderate scores of innovation, high probability of implementation as well as moderate to high impact on older people with frailty which referred to the model’s ability to solve current care delivery issues such as lack of guidelines and accountability for care management. None of the system-based integrated care models were regarded as particularly innovative and all had moderate impact on the older people with frailty. In terms of the community-based interventions, EuFrailSafe had the overall highest scores with high scores on all three categories. None of the community-based interventions scored low in any category.

#### Qualitative assessment

The quantitative scores were further substantiated by qualitative assessments where the participants commented on how the five system-based integrated care models and four community-based interventions could help solve system fragmentation issues (online supplemental table 2). The participants stated how innovative service delivery approaches targeted at the older people with frailty should involve these themes: (1) an assigned frail coordinator, (2) integrated patient information systems, (3) multidisciplinary teamwork, (4) competency within frailty, (5) patient and network empowerment, as well as (6) a shift from specialist acute reactive care to primary preventative, proactive care.

For example, the system-based WICM model was seen to be favourable due to its focus on community care, teamwork and caregiver involvement. However, despite the consensus among participants that certain traits of system-based integrated care models were considered vital for delivering holistic care (ie, caregiver support in Program of All-Inclusive Care for the Elderly and Geriatric Resources for Assessment and Care of Elders, proactive detection for frailty in WICM and a frailty coordinator in Program of Research to Integrate the Services for the Maintenance of Autonomy and Services intégrés pour les personnes âgées fragiles), there was uncertainty about how they would be adapted and applied in the Norwegian context.

The participants viewed community-based interventions focusing on welfare technology (EuFrailSafe), active social network participation (integrated neighbourhood approach), comprehensive home care services (Hospital at Home), and frailty education (massive open online courses) as both in line with frailty care needs and trends as well as easily adaptable to the Norwegian environment. The use of technological devices, such as described in the EuFrailSafe model, was highlighted as innovative.

### Horizon scanning as a decision-making tool

Horizon scanning, according to the participants, could be a valuable decision-making tool as it involved assessing knowledge gaps, criticising the status quo, developing new insights on the topic of concern and networking with experts prior to the implementation of measures.

It is a method for gaining more knowledge and translating it into practice with expert assessments. It can be a way to collaborate with other knowledge communities, once you have identified an information gap. (Participant 2)

In addition, the participants emphasised that the method would necessitate expertise and should be carried out by policymakers to shed light on possible implementational challenges.

The method requires good systematic literature search. That is the foundation of the process. Not everyone can do that. The filtering and prioritisation criteria are choices one needs to make and if unsure, the process can give the wrong results. It is a subject of its own, so it has to be done at a higher organisational level. (Participant 5)

The participants expressed that the results of the horizon scan were challenging to comprehend and evaluate.

These models are complex, and it is difficult to get an overall understanding of them. (Participant 4)

## Discussion

In line with the study’s objectives, the small-scale horizon scan conducted in this study identified novel integrated care models and interventions, the majority of which were regarded by the participants as innovative, had the potential to impact the older people with frailty and were appropriate to some degree, for implementation in the Norwegian healthcare system. Additionally, the discussion of models and interventions was able to give the participants insight into needs and trends of integrated care as well as alternative solutions to address information gaps, system fragmentation and current service innovation.

However, participants raised some concerns about the potential adaptability and applicability of the system-based integrated care models to a Norwegian context. This finding is not surprising. Studies of integrated care models suggest that the higher the level of integration specified by the design, the higher the level of differentiation.^52 53^ In Norway, integrated care involving different decision-making levels is hindered by lack of formalised coordination and cooperation between hospitals and municipalities.^21^ Thus, in this setting, the various components of integration present in the system-based models necessitate large-scale changes in legal and financial regulations, as well as organisational reorganisation and thus, government support for implementation would be required.

On the other hand, the participants gave high scores to the more discrete interventions focusing on specific components of integrated care at the community level. As many of the participants held municipal-level positions, it may have been easier for them to envision how these interventions could be implemented without requiring major legislative changes.

In this study, it was assumed that criteria such as potential for impact, innovativeness and implementation are equally weighted. It is important to note that the scores can be changed as policymakers and healthcare authorities may weigh these criteria differently based on the country’s healthcare goals.^54^

According to the participants, horizon scanning was deemed a beneficial tool to employ as it entailed assessing knowledge gaps, questioning the status quo, getting new perspectives on approaching the topic of concern and networking with other experts prior to implementing interventions. However, there were varying opinions on the process’s practical application. This uncertainty may be due to the study’s participants having little to no prior knowledge of horizon scanning and its use in decision-making. Involvement from participants from the beginning of the search process rather than simply during the assessment phase may be necessary to ensure that the participants receive adequate time to comprehend, reflect on and analyse the methodologies’ practical consequences. Participants also expressed support for the creation of a central decision-making body to carry out horizon scanning of novel healthcare service models and interventions.

Since horizon scanning is a systematic methodology, it may require that the horizon scanner(s) have some level of competency in performing accurate literature searches on the topic of concern. This would imply that prior to the search process, the horizon scanner(s) are aware of the information gaps that need to be filled in accordance with national healthcare priorities and that the horizon scanner(s) may need access to input from national decision-makers to shed light on potential implementation challenges such as resource implications, cooperation of stakeholders, ethical and accessibility issues. This could be seen as an essential step for establishing database selection, filtration and prioritisation criteria that would be able to guide the extensive search process and prevent the removal of relevant records of information that meet the stakeholders’ needs.^55^

Horizon scanning may be performed by relying solely on secondary sources of data, as demonstrated in this study. However, to increase the probability of attaining ‘new and emerging’ results from a horizon scan, the methodologies may require access directly from policymakers and healthcare authorities (primary source) to restricted information on models and interventions that are still under development but have not yet been published. Moreover, access to specialised databases of horizon scanning organisations (tertiary source) that can help with search optimisation would be beneficial.^56^

### Limitations

Current horizon scanning guidelines from EuroScan and the NHSC directed towards pharmaceuticals and health technologies were used in this study.^34 56^ Even though the guidelines were adapted to fit the study’s objectives and ensure validity, these guidelines are generally used to target the early lifecycle of technologies. Healthcare services, such as integrated care models and interventions, are often already developed and established as practices in a given setting when discussed in the literature or in other sources of information. Thus, we found it difficult to scan for ‘new’ initiatives in this context, although they were new to a Norwegian setting.

At the same time, horizon scanning should not be regarded as a systematic literature review.^57^ Signals of ‘things to come’ are detected from manifold information sources in addition to, or even instead of, reviews of scientific literature. Thus, horizon scanning can lack a clear weighting of evidence and should not be misinterpreted to give an exhaustive summary of current evidence. The aim of horizon scanning is rather to inform decision-makers about signs of innovation at an early stage, at which point available information, including information about intervention effect, is limited.

Even though we used guidelines, we cannot rule out the possibility that bias was introduced into the scanning’s filtration and prioritisation process. During the focus group session, considerations were taken with regard to minimising the moderator’s facilitation of conversation, encouraging the development of independent viewpoints so that the participants could challenge one another, avoid groupthink and not be easily influenced by a dominant voice. This was done in addition to sending out the topic guide prior to the session. However, because the participants had limited prior knowledge and potentially a lack of time to establish a good understanding of the horizon scanning methodologies and the nine models and interventions, a limitation of this study could be the reliability of the participants’ assessment. With hindsight, the participants should have been given more time in the focus group.

The transferability of the results to other settings may be limited. We carried out a small-scale horizon scanning review with a small sample size, even though each participant had multiple roles in various work settings. This limits the validity of the results through increased bias. In a more comprehensive study, several measures could be taken to improve the validity of the results. For example, a Delphi technique could have been used, with an anonymous review, scoring and commenting, before a focus group discussion.^58^ Moreover, involvement of different stakeholder groups, such as policymakers, public and patients, could have been included in the assessment and prioritisation of possible interventions. While the focus group session was in depth, involving diverse stakeholders such as patients and their caregivers as well as increasing the number of participants may have improved the breadth of findings. In addition, conducting multiple focus groups where the models, interventions and horizon scanning methodologies could be discussed and evaluated more comprehensively until no new knowledge is gained from subsequent sessions (saturation) may have strengthened the reliability of the assessments.^58^

## Conclusion

By using a horizon scanning methodology, new and emerging integrated care models and interventions for the older people with frailty which have the potential to overcome system fragmentation and enhance care coordination have been identified. Furthermore, the horizon scanning process enabled discussion on the need for integrated care and the perceived difficulties of implementing the discussed models and interventions in the Norwegian context. In doing so, horizon scanning may be seen as a valuable tool policy decision-makers and healthcare authorities may use for tackling information gaps and creating innovation in service delivery. Further research should look at how the horizon scanning process could be carried out in a real-world environment.

## Supplementary Material

Reviewer comments

Author's manuscript

## Data Availability

Data are available upon reasonable request.
