# Supplementary material for: Models of integrated care for older people with frailty: a horizon scanning review
Source: BMJ Open. 2022 Apr 8;12(4):e060142. doi: 10.1136/bmjopen-2021-060142 (PMC8996021; doi:10.1136/bmjopen-2021-060142)
Supplement: Supplementary data [file bmjopen-2021-060142supp003.pdf]

Supplemental material

Supplementary table 1: Backgrounds of participants

**Regional health authority**	**N**	**Background of participant**
Central Norway	4	Senior researcher/ Nurse/ Professor<br>Physiotherapist/ Project manager<br>Advisor<br>Senior Advisor/ Phd-fellow/ Nurse
West	1	Research coordinator/ Nurse specialist (oncology)
South-East	2	Specialist in internal medicine and geriatrics/ Chief physician/ Professor<br>Leader of community services development/ Nurse
Total	7	

Supplementary figure 1: Interview guide

"Horizon scanning of healthcare delivery models within services targeted to the frail elderly"

Focus group agenda

Overall aim of this study

To evaluate if horizon scanning can be used to help decision makers to fill in the knowledge gaps, address issues such as system fragmentation, as well as contribute to innovation of the healthcare delivery services targeted at the frail elderly.

Program

No.	Topic	Focus	Time
1.	Introduction	- Brief description of the study's purpose<br>- Participants' presentation of themselves	1530-1540
2.	Horizon scanning process	- Description of horizon scanning steps<br>- Presentation of results from the scan	1540-1550
3.	System-based models	- Introduction of each model<br>- Discussion<br>- Evaluation	1550-1630
4.	Community-based interventions	- Introduction of each intervention<br>- Discussion<br>- Evaluation	1630-1650
5.	Horizon scanning methodology	- Discussion and evaluation	1650-1700

Interview guide

Do you have any potential conflicts of interest, such as ongoing research or other intellectual / financial interests with organizations related to the models/interventions discussed in this interview?

Yes No

If yes, please describe:

List of innovations

To avoid miscommunication we define new innovations as a possible new way of organizing services, a new mechanism in the service process, changes in the system that increase access to more comprehensive services for frail elderly as well as a new application of existing intervention (s), or other current innovations.

The list is structured after system-based and community-based with an aim to create a better overview for discussion and evaluation. The division is not unambiguous as the integrated care models and community-based interventions do contain overlapping elements.

The innovations placed under "system-based" contain core traits of integrated care models specific for frail elderly on a system/population large scale level.

The innovations placed under "community-based" contain traits that allow for the frail to live independently in the community. These have a "door in" approach and are on a more local/community small scale level. This does not mean the community-based are not involved in system level decision making and vice versa.

Discussion & Evaluation on list of innovations

Please reply if you are aware of the mentioned innovations, and, if applicable, leave a comment on the various innovations.

System-based Innovation	Do you know this?	Additional comments
PRISMA	Yes/No	
SIPA	Yes/No	
WICM	Yes/No	
PACE	Yes/No	
GRACE	Yes/No	

Community-based Innovation	Do you know this?	Additional comments
EuFrailSafe	Yes/No	
INA	Yes/No	
MOOCs	Yes/No	
Hospital at Home	Yes/No	

Based on the description and your experience, please rate them on a scale of *low, moderate, and high* accordingly to

i) Level of innovation: degree of novelty, filtration of services from that of common practice.

ii) Probability that the innovation will be further implemented in the next 2-10 years: to see which innovations most likely to be in the horizon of integrated healthcare services for frail elderly. *Things to consider here are resource implications, expected utilisation and availability of the innovation across different geographical areas, actions required before implementation can take place, time, and investment in training of personnel, cooperation of stakeholders and ethical and accessibility issues.*

iii) Likely impact on frail elderly: importance/quality of the innovation. *Things to consider here are the innovation's ability to solve current service issues such as disease-focused treatments, long waiting times, poor exchange of knowledge/collaboration among health workers as a result of not having a shared electronic health record, insufficient staff numbers, lack of guidelines and accountability for care management, absence of professional expertise regarding the patient's health condition, lack of clarity with regards to health personnel's duties and responsibilities as well as a failure in offering updates to patients and their families, along with preparing them for future care transfers.*

System-based Innovation	Level of innovation	Comments	Probability of implementation in the next 2-10 years	Comments	Likely impact on frail elderly	Comments
PRISMA						
SIPA						
WICM						
PACE						
GRACE						

Community-based Innovation	Level of innovation	Comments	Probability of implementation in the next 2-10 years	Comments	Likely impact on frail elderly	Comments
EuFrailSafe						
INA						
MOOCs						
Hospital at Home						

Discussion & Evaluation on horizon scanning methodology

What are the current methods you use for making decisions in healthcare service delivery?

What do you think of horizon scanning as a tool for decision making in healthcare service delivery?

What would be the possible strengths and weaknesses of using such a tool?

Any further comments?

Supplementary table 2: Illustrative quotes from Qualitative assessment

**Shifting away from specialist acute reactive care**	**EuFrailSafe**<br>"It is a trend and a need to focus on prevention with the use of technology" Informant 5	**Hospital at Home**<br>"If it is well organised within the municipality and we are familiar with the patient's background and medical issues, then it is best and definitely possible to treat them at home" Informant 4	**MOOCs**<br>"This seems to be innovative as it is prevention focused and more customised for frail people and their caregivers, plus the information is easily accessible" Informant 3	**WICM**<br>"I think it would be beneficial if the frail elderly patients were screened early at the doctor's office to avoid hospital admissions" Informant 6
**Silos**	**PRISMA & SIPA**<br>"I like that there is a defined team responsible for the patient's cate and the focus is on coordination" Informant 3	**INA**<br>"A social worker who acts as a coordinator and does assessments at the frail person's home while involving neighbours and volunteers is new and innovative. I have never heard about it" Informant 7		
**Service gaps and duplications**	**EuFrailSafe**<br>"The virtual platform and use if monitoring devices allow for better clinical follow-up and care" Informant 6	**PRISMA & SIPA**<br>"The connection between the specialist service and the primary health service is poor, we do not have any communication while in these models, there is a team and a platform they use to meet and plan and the care for the patient. I think it is a great idea especially for the frail with complex health problems" Informant 7	**GRACE & PACE**<br>"These models seem to have good collaboration routines between the specialist and the primary health services as well as interdisciplinary teams within the primary health service which I feel is important" Informant 3	
**Competence requirements**	**MOOCs**<br>"I think it would be useful as you get knowledge about the health condition you are struggling with and support from others" Informant 5	**WICM**<br>"The idea of a nurse practitioner and family physician teaming up to do geriatric assessments for frailty and early deterioration among elderly is innovative" Informant 1		
**Greater patient and network involvement**	**WICM**<br>"There is active caregiver support and involvement, that is innovative" Informant 1	**GRACE & PACE**<br>"I believe that in the future with the lack of healthcare personnel and a growing number of elderlies, initiatives that involve the	**INA**<br>"It would be useful and something we would need in the future as there is a focus on strengthening social networks in a local	

		network surrounding the frail patient will become essential" Informant 5	environment, where a neighbourhood takes responsibility for the care of the frail" Informant 2	
