# Supplementary material for: Models of integrated care for older people with frailty: a horizon scanning review
Source: BMJ Open. 2022 Apr 8;12(4):e060142. doi: 10.1136/bmjopen-2021-060142 (PMC8996021; doi:10.1136/bmjopen-2021-060142)
Supplement: Author's manuscript [file bmjopen-2021-060142.draft_revisions.pdf]

BMJ Open

BMJ Open is committed to open peer review. As part of this commitment we make the peer review history of every article we publish publicly available.

When an article is published we post the peer reviewers' comments and the authors' responses online. We also post the versions of the paper that were used during peer review. These are the versions that the peer review comments apply to.

The versions of the paper that follow are the versions that were submitted during the peer review process. They are not the versions of record or the final published versions. They should not be cited or distributed as the published version of this manuscript.

BMJ Open is an open access journal and the full, final, typeset and author-corrected version of record of the manuscript is available on our site with no access controls, subscription charges or pay-per-view fees (http://bmjopen.bmj.com).

If you have any questions on BMJ Open's open peer review process please email info.bmjopen@bmj.com

BMJ Open

Models of integrated care for older people with frailty: A horizon scanning review

Journal:	*BMJ Open*
Manuscript ID	bmjopen-2021-060142
Article Type:	Original research
Date Submitted by the Author:	12-Dec-2021
Complete List of Authors:	A Kjelsnes, Ashwanee; University of Oslo,<br>Feiring, Eli; University of Oslo,
Keywords:	Health policy < HEALTH SERVICES ADMINISTRATION & MANAGEMENT, Organisation of health services < HEALTH SERVICES ADMINISTRATION & MANAGEMENT, HEALTH SERVICES ADMINISTRATION & MANAGEMENT

SCHOLARONE™
Manuscripts

I, the Submitting Author has the right to grant and does grant on behalf of all authors of the Work (as defined in the below author licence), an exclusive licence and/or a non-exclusive licence for contributions from authors who are: i) UK Crown employees; ii) where BMJ has agreed a CC-BY licence shall apply, and/or iii) in accordance with the terms applicable for US Federal Government officers or employees acting as part of their official duties; on a worldwide, perpetual, irrevocable, royalty-free basis to BMJ Publishing Group Ltd ("BMJ") its licensees and where the relevant Journal is co-owned by BMJ to the co-owners of the Journal, to publish the Work in this journal and any other BMJ products and to exploit all rights, as set out in our licence.

The Submitting Author accepts and understands that any supply made under these terms is made by BMJ to the Submitting Author unless you are acting as an employee on behalf of your employer or a postgraduate student of an affiliated institution which is paying any applicable article publishing charge ("APC") for Open Access articles. Where the Submitting Author wishes to make the Work available on an Open Access basis (and intends to pay the relevant APC), the terms of reuse of such Open Access shall be governed by a Creative Commons licence – details of these licences and which Creative Commons licence will apply to this Work are set out in our licence referred to above.

Other than as permitted in any relevant BMJ Author's Self Archiving Policies, I confirm this Work has not been accepted for publication elsewhere, is not being considered for publication elsewhere and does not duplicate material already published. I confirm all authors consent to publication of this Work and authorise the granting of this licence.

For peer review only

Models of integrated care for older people with frailty: A horizon scanning review

1 Ashwanee A

Department of Health Management and Health Economics

PO Box 1089 Blindern

0317 Oslo

Norway

Mail: ashwanee.kjelsnes@studmed.uio.no

Orchid id: https://orcid.org/0000-0001-7300-3614

2 Eli Feiring

Department of Health Management and Health Economics

PO Box 1089 Blindern

0317 Oslo

Norway

Orchid id: https://orcid.org/0000-0001-5280-1051

Word count (4123)

For peer review only

ABSTRACT

Objectives Frailty, a multifaceted geriatric condition, is an emerging global health problem. Integrated care models designed to meet the complex needs of the older people with frailty are required. Early identification of innovative models may inform policymakers and other stakeholders of service delivery alternatives they can introduce and locally adapt so as to tackle system fragmentation and lack of coordination. This study used horizon scanning methodologies to systematically search for, prioritise and assess new integrated care models for older people with frailty and investigated experts' views on barriers and facilitators to the adoption of horizon scanning in health services research.

Methods A four-step horizon scanning review was performed. Frailty specific integrated care models and interventions were identified through a review of published literature supplemented with grey literature searches**.** Results were filtered and prioritised according to pre-set criteria. An expert panel focus group session assessed the prioritised models and interventions on innovativeness, impact and potential for implementation. The experts further evaluated horizon scanning for its perceived fruitfulness in aiding decision-making.

Results Nine integrated care models and interventions at system level (n=5) and community level (n=4) were summarised and assessed by the expert panel (n=7). Test scores were highest for the Walcheren integrated care model (system-based model) and EuFrailSafe (community-based intervention).The participants stated that horizon scanning as a decision making tool, could aid in assessing knowledge gaps, criticising the status quo and developing new insights. Barriers to adoption of horizon scanning on individual, organisational and wider institutional level were also identified.

For peer review only - http://bmjopen.bmj.com/site/about/guidelines.xhtml

Conclusion Study findings demonstrated that horizon scanning is a potentially valuable tool in the search for innovative service delivery models. Further studies should evaluate how horizon scanning can be institutionalised and effectively used for serving this purpose.

Strengths and limitations of this study

- This horizon scanning review identified promising models of integrated care for the older people with frailty by a process of information mapping, filtration, prioritisation, and assessment.
- Facilitators and barriers to using horizon scanning were identified and should be taken into consideration when discussing adopting this method in the context of health service delivery models.
- Due to the amount and complexity of information, focus group participants may not comprehensively have understood all models and interventions presented to them in the short time available.

For peer review only

INTRODUCTION

Frailty, a multifaceted geriatric condition characterised by increased vulnerability to stress incidents due to reductions in reserve and functions in multiple physiological systems, is emerging as a global health problem with significant clinical and public health consequences,[1-4]. It is approximated that 21.3 percent of the world's population will be 60 or older by 2050, where frailty is estimated to affect around one out of every six community-dwelling seniors,[5]. Frailty is associated with a significant increase in comorbid chronic illnesses, functional dependency, disability, healthcare needs and costs,[6-7]. To avoid or delay the progression of frailty to significant functional decline, healthcare designed to meet the complex care requirements is needed,[1, 8-11]. In Norway, as in many other countries, establishing high-quality integrated care for older people with frailty is a political priority,[12]. Integrated care, understood as comprehensive, multi-level and across settings organisation of care, is generally believed to be a solution to the demand for improved care for the multimorbid and long-term care patients,[13]. However, a recent systematic review on integrated care models for managing and preventing frailty concluded that few models were specifically designed to prevent and tackle frailty in the community and at the interface between primary care and secondary care,[14].

The absence of a standardised frailty definition and assessment method coupled with the fact that literature on frailty specific integrated care models and interventions are still in their early stages of development makes it challenging for healthcare decision makers to meet the needs of the older people with frailty,[15-17]. The search for signals of important development in this context can possibly be lessened by horizon scanning, which acts as an information resource that can aid in decisions about the identification of innovative health-care interventions,[18].

For peer review only - http://bmjopen.bmj.com/site/about/guidelines.xhtml

Horizon scanning is a systematic approach for detecting early signals of potentially important developments that could impact areas of interest,[19]. It involves a comprehensive review of data to bridge knowledge gaps, question assumptions, assess possible threats, challenges and emerging problems, as well as look for opportunities to present new policy alternatives,[20-23]. Signals of "things to come" are detected from manifold information sources in addition to, or even instead of, reviews of scientific literature. These sources include targeted literature searches and input from expert groups, committees, surveys, government bodies, conferences, associations, media and more. Further, experts and other stakeholders with diverse views, experiences, and roles may be brought together to systematically discuss signals as part of the horizon scanning process.

In healthcare, horizon scanning methodologies are commonly used as a health technology assessment (HTA) tool in early awareness and alert (EAA) systems of pharmaceuticals to allow for innovative medicines to enter the market. Less attention has been given to the employment of horizon scanning methodologies in identifying improvements for delivery of healthcare services,[24].

At this backdrop, we wanted to investigate if employing horizon scanning methodologies could be a valuable and viable strategy to identifying novel integrated care initiatives for older people with frailty, in an early phase of adoption. First, we aimed to identify new and emerging integrated care models and interventions that could potentially address system fragmentation issues faced by the older people with frailty and use the opinions of experts to evaluate these models and interventions based on their level of innovation, probability of implementation and impact.

For peer review only - http://bmjopen.bmj.com/site/about/guidelines.xhtml

The second aim was to look into experts' opinions on the fruitfulness of employing horizon scanning methodologies in this context, given horizon scanning is still a relatively new tool for identifying innovative healthcare delivery models.

METHODS

Study design

This study was designed as a small-scale horizon scanning.

The PRISMA guidelines were used to report the literature search process as far as possible, and the COREQ guidelines were used to report the findings from the qualitative focus group (supplementary file 1 and 2). The study was notified to and assessed to be in accordance with relevant guidelines by the Norwegian Centre for Research Data (project number: 948039).

Setting

The Norwegian healthcare system is universal, tax-financed, and semi-decentralised,[25]. The responsibility for primary health and social care lies with the municipalities. The central state is responsible for secondary and specialist health care, which is administrated by four Regional Health Authorities (RHAs). The lack of communication between the two tiers of governance contributes to challenges with delivery of integrated care,[26]. Although a Coordination Reform (2012) established mandatory network governance to improve coordination between primary and specialist care, integrated care involving different levels is hindered by lack of formalised coordination and cooperation between the municipalities and the hospitals,[12].

For peer review only

Horizon scanning to identify novel integrated care models

Horizon scanning generally follows a six-step approach of signal detection, filtration, prioritisation, assessment, and dissemination and updating information (figure 1). The first step includes mapping signals of innovation with the use of literature reviews, including reviews of grey literature and reports retrieved from governmental bodies, conferences, meetings, forums, observatories, and other organisations. Pre-set filtration and prioritisation criteria are used to discard irrelevant signals. Assessment methods include participation of experts, users and policymakers, and peer reviews. The results of the horizon scanning are then disseminated and evaluated,[24].

A horizon scanning may be carried out at the beginning of a broader foresight process, aiming to address the full cycle of policy on "complex futures" and involving a range of stakeholders, long-term considerations and different scenarios. It may, however, also be a stand-alone approach for identifying "things to come". In the present study, the horizon scanning process carried out followed the first four steps of the EuroScan methods toolkit for early awareness and alert systems (EAAS),[24]. We conducted a focus group session to obtain thoughts on integrated care needs for older people with frailty, as well as opinions on the models and interventions identified in the literature and perspectives on horizon scanning methodologies and its potential consequences.

We followed a multifaceted definition of "integrated care" in this study. Integrated care models can be organised according to target group, level and degree (figure 2). Thus, we kept a broad understanding of integrated care as an organisational coordination mechanism that can be understood as to providing a cohesive and continuum of care that is personalised to the patient's condition,[27-29].

For peer review only

Figure 1

Figure 2

Literature search strategy: Identification, filtration and prioritisation of records

Search strategy

Reviews of published literature and grey literature were performed to trace new and emerging integrated care models and interventions, targeted at the older people with frailty, which had the potential in addressing system fragmentation issues. Databases and governmental bodies were searched using pre-specified search terms to identify research papers, proceedings of conferences and workshops, policy papers and reports. Only records published in English or Norwegian were included. The final search took place from 01.11.2020 to 01.02.2021.

Information sources	Search terms
Online databases<br><br>• Pubmed (384)<br>• Cochrane Library (19)<br>• Evidence-based medical reviews (24)<br>• Embase (349)<br>• Oria UiO (50)<br>• JStor (92)<br>• Medline Ovid (27)<br>• Web of Science (41)	• Frail elderly<br>• Integrated care model<br>• Multidisciplinary<br>• Aged care<br>• Service delivery model<br>• Older people<br>• Geriatric<br>• >65<br>• Health sciences

For peer review only - http://bmjopen.bmj.com/site/about/guidelines.xhtml

• Scopus (104)	• Political sciences<br>• Public health<br>• Public policy and administration<br>• Health policy
Governmental reports and conferences<br><br>• Norwegian institute of public health (20)<br>• The Norwegian Directorate of Health (29)<br>• Ministry of Health and Care Services (10)<br>• Norwegian National Advisory Unit on Ageing and Health (28)<br>• The innovation conference: the outward-looking hospital (1)<br>• Frailty among the elderly conference (1)	

Table 1 Information sources and search terms used for signal detection

Inclusion and exclusion criteria

Findings were filtered by scanning each record's abstract, title and keywords based on a set of inclusion and exclusion criteria, which were adapted from EuroScan,[24] and the National Horizon Scanning Centre (NHSC) guidelines for horizon scanning,[30] as well as from previous literature. Records that dealt with the adoption, execution, or assessment of initiatives focused on the concept of patient-centred integration: "funding, administrative, organisational, service delivery, and clinical levels required to promote interaction, coordination, and cooperation in and between the cure and care sectors were included,[15].

Records focused solely on integrated care, multidisciplinary team and frailty without describing any intervention and/or model, as well as those not specifically focused on the

For peer review only - http://bmjopen.bmj.com/site/about/guidelines.xhtml

older people with frailty, were excluded. Disease-specific publications were removed because frailty is considered a multi-faceted and dynamic disease,[31-38].

Remaining records were then grouped into system-level integrated care models and community-based interventions with an aim to create a better overview for discussion and evaluation. The grouping was not unambiguous as the integrated care models and community-based interventions do contain overlapping elements. We included records that described models that had some or all of the characteristics illustrated in Table 2.

Prioritisation of models and interventions prior to focus group assessment

Prior to focus group assessment, we did a criteria-informed qualitative prioritisation of the system-based models and community-based interventions (Table 2). The aim of the prioritisation was to identify models and interventions not yet implemented or tested in a Norwegian setting, which we considered to have the potential to address system fragmentation issues.

System-level integrated care models for older people with frailty	Community-based interventions for older people with frailty.	Prioritisation criteria
• Centralised point of entry<br>• Geriatric evaluations<br>• Case management<br>• Multidisciplinary teams	• Local or community level-based interventions<br>• Living-at-home<br>• Measures described to promote independence	• Potential care outcomes<br>• Potential cost-effectiveness<br>• Expected resource utilisation

For peer review only - http://bmjopen.bmj.com/site/about/guidelines.xhtml

• Multidisciplinary guidelines and meetings<br>• Digitalised patient files<br>• Network framework		• Expected reorganisation of services<br>• Applicability<br>• Novelty<br>• Forward thinking

Table 2 Intervention characteristics and considerations used to filter and prioritise models and interventions,[34] ,[39-40].

Focus group: Assessment of records

Participants and recruitment

The focus group's goal was to discuss and assess the literature review's findings. Purposive sampling was used to recruit participants that had a variety of roles and educational backgrounds as well as knowledge of services provided to the older people with frailty,[41]. The research team approached the Norwegian National Advisory Unit on Ageing and Health and was set in contact with potential participants that were subsequently invited to the study. The invitees further provided potential participants (snowball sampling). Eleven persons were invited to participate.

Data collection

The focus group was conducted on 07.04.2021 via Zoom by AA. Consent forms were signed and collected prior to the focus group.

Prior to the focus group discussion, the participants were emailed information on the horizon scanning process conducted, tables of the identified models and interventions, as well as the

For peer review only - http://bmjopen.bmj.com/site/about/guidelines.xhtml

semi-structured topic guide (supplemental material figure 1). They were asked to score and evaluate the different models independently, but we did not collect their evaluations before the focus group took place. This was a pragmatic choice given the study's time- and resource limits.

The focus group session was divided into three sections. The first section presented a summary of the horizon scanning process as well as the key features of each model and intervention. This was done to clear up any misunderstandings or questions they had about the models and horizon scanning process. The models and interventions were organised and presented in accordance with the various forms of integration, with the aim of demonstrating how they provided complex integrated care to the frail in a clear and understandable manner. To avoid miscommunication among the participants, "innovations" were defined as i) as a possible new way of organising services, ii) a new mechanism in the service process, iii) changes in the system that increase access to more comprehensive services for older people with frailty, iv) a new application of existing intervention(s), or other current innovations,[42].

The second section focused on assessment of the models and interventions where the participants were asked to collectively discuss, reflect and rate each model and intervention on a scale from low to high, on the following equally-weighted aspects; level of innovation, probability for implementation in the next 2-10 years, and potential impact on the older people with frailty. Further details of what these three aspects meant were also included in the interview guide (supplemental material figure 1). Participants were finally asked to offer their thoughts on horizon scanning, its prospective implications and potential for use as a decision-making tool.

For peer review only - http://bmjopen.bmj.com/site/about/guidelines.xhtml

The focus group session lasted two hours. Discussions were recorded on a password-protected computer connected to a university server. The transcription was done through coding to protect the anonymity of the participants.

Data analysis

Organisation and analysis of data collected from the focus group discussion followed the continuum of data analysis framework,[43]. Data were transcribed and organised according to the topic guide ensuring that both positive and negative comments with regards to each model and intervention evaluated against the three criteria, were included. The descriptive statements were then indexed, arranged, compared, analysed and rearranged to create categories for both quantitative and qualitative results. Data used as illustrative purposes were translated from Norwegian to English by the authors.

Patient or public involvement

Patients and public were not involved in any part of our research.

RESULTS

Identification, filtration and prioritisation

There were 1179 records identified through the initial database searches and grey literature, of which 605 were removed due to failing to meet the inclusion criteria at the filtration stage. One hundred and fifty-five duplicates and 134 disease-specific records were excluded, and 181 records were thereafter removed after reading through the full text for relevance. At the prioritisation stage, one hundred and four records were read and evaluated according to the prioritisation criteria. Nine records were included in this study after prioritisation (figure 3). Figure 4 gives an overview over the models and interventions detailed by the records.

For peer review only - http://bmjopen.bmj.com/site/about/guidelines.xhtml

Figure 3

Five system-based models and four community-based interventions were prioritised to be assessed in the focus group (figure 4). These models and interventions were to be applied at different key points in the frailty care pathway,[44] such as preventive education, enablement and care and support at home, assessment at management in primary care, geriatric assessment in hospital and intermediate care services,[44]. While the system-based models are developed to give comprehensive integrated chronic care, the community-based interventions are more discrete interventions that provide specific components of integrated care .

Figure 4

Evaluation

Participants

Eleven persons were invited to participate in the focus group; four declined the invitation due to other work commitments. The seven participants that took part were experienced healthcare professionals with various educational backgrounds and had multiple roles in academia, specialist and primary care. They resided in different parts of the country (supplemental material table 1).

Quantitative scores

The participants discussed and then agreed on a score for each system- level integrated care models and community-based interventions together on the three aspects: innovation, implementation, and impact on a low, moderate, and high scale. The scores are stated below in Table 3.

For peer review only
For peer review only - http://bmjopen.bmj.com/site/about/guidelines.xhtml

System-based integrated care model	Level of innovation	Probability of implementation in the next 2-10 years	Likely impact on frail elderly
PRISMA	L	L/M	M
SIPA	L	L/M	M
WICM	L/M	H	M/H
PACE	L/M	L	M
GRACE	L/M	L	M

Community-based intervention	Level of innovation	Probability of implementation in the next 2-10 years	Likely impact on frail elderly
EuFrailSafe	H	H	H
INA	H	M	M/H
MOOCs	M/H	M/H	M
Hospital at Home	M	M/H	M/H

Table 3 Scoring of models and interventions

The Walcheren Integrated Care Model (WICM) had the overall highest scores among the system-based integrated care models. It received low to moderate scores of innovation, high probability of implementation as well as moderate to high impact on older people with frailty which referred to the model's ability to solve current care delivery issues such as lack guidelines and accountability for care management. None of the system-based integrated care models were regarded as particularly innovative and all had moderate impact on the older people with frailty. In terms of the community-based interventions, EuFrailSafe had the overall highest scores with high scores on all three categories. None of the community-based interventions scored low in any category.

Qualitative assessment

The quantitative scores were further substantiated by qualitative assessments where the participants commented on how the five system-based integrated care models and four community-based interventions could help solve system fragmentation issues (supplemental material table 2). The participants stated how innovative service delivery approaches targeted

For peer review only - http://bmjopen.bmj.com/site/about/guidelines.xhtml

at the older people with frailty should involve these themes, (i) an assigned frail coordinator, (ii) integrated patient information systems, (iii) multidisciplinary teamwork, (iv) competency within frailty, (v) patient and network empowerment as well as a (vi) shift from specialist acute reactive care to primary preventative, proactive care.

For example, the system-based WICM model was seen to be favourable due to its focus on community care, teamwork, and caregiver involvement.

However, despite the consensus among participants that certain traits of system-based integrated care models (i.e., caregiver support in PACE and GRACE, proactive detection for frailty in WICM and a frailty coordinator in PRISMA and SIPA) were considered vital for delivering holistic care, there was uncertainty about how they would be adapted and applied in the Norwegian context.

The participants viewed community-based interventions focusing on welfare technology (EuFrailSafe), active social network participation (INA), comprehensive home care services (Hospital at Home), and frailty education (MOOCs) as both in line with frailty care needs and trends as well as easily adaptable to the Norwegian environment. The use of technological devices, such as described in the EuFrailSafe model, was highlighted as innovative.

Horizon scanning as a decision-making tool

Horizon scanning, according to the participants, could be a valuable decision-making tool as it involved assessing knowledge gaps, criticising the status quo, developing new insights on the topic of concern, and networking with experts prior to the implementation of measures.

> *It is a method for gaining more knowledge and translating it into practice with expert assessments. It can be a way to collaborate with other knowledge communities, once you have identified an information gap* Participant 2.

For peer review only - http://bmjopen.bmj.com/site/about/guidelines.xhtml

In addition, the participants emphasised that the method would necessitate expertise and should be carried out by policymakers to shed light on possible implementational challenges.

> *The method requires good systematic literature search. That is the foundation of the process. Not everyone can do that. The filtering and prioritisation criteria are choices one needs to make and if unsure, the process can give the wrong results. It is a subject of its own, so it has to be done at a higher organisational level* Participant 5.

The participants expressed that the results of the horizon scan were challenging to comprehend and evaluate.

> *These models are complex, and it is difficult to get an overall understanding of them* Participant 4.

DISCUSSION

In line with the study's objectives, the small-scale horizon scan conducted in this study identified novel integrated care models and interventions, the majority of which were regarded by the participants as innovative, had the potential to impact the older people with frailty and were appropriate to some degree, for implementation in the Norwegian healthcare system. Additionally, the discussion of models and interventions were able to give the participants insight into needs and trends of integrated care as well as alternative solutions to address information gaps, system fragmentation and current service innovation.

However, participants raised some concerns about the potential adaptability and applicability of the system-based integrated care models to a Norwegian context. This finding is not surprising. Studies of integrated care models suggest that the higher the level of integration specified by the design, the higher the level of differentiation,[54-55]. In Norway, integrated care involving different decision-making levels is hindered by lack of formalised coordination

For peer review only - http://bmjopen.bmj.com/site/about/guidelines.xhtml

and cooperation between hospitals and municipalities,[21]. Thus, in this setting, the various components of integration present in the system-based models necessitate large-scale changes in legal and financial regulations, as well as organisational reorganisation and thus, government support for implementation would be required.

On the other hand, the participants gave high scores to the more discrete interventions focusing on specific components of integrated care at the community level. As many of the participants held municipal-level positions, it may have been easier for them to envision how these interventions could be implemented without requiring major legislative changes.

In this study, it was assumed that criteria such as potential for impact, innovativeness and implementation are equally weighted. It is important to note that the scores can be changed as policymakers and healthcare authorities may weigh these criteria differently based on the country's healthcare goals,[56].

According to the participants, horizon scanning was deemed a beneficial tool to employ as it entailed assessing knowledge gaps, questioning the status quo, getting new perspectives on approaching the topic of concern, and networking with other experts prior to implementing interventions. However, there were varying opinions on the process's practical application. This uncertainty may be due to the study's participants having little to no prior knowledge of horizon scanning and its use in decision making. Involvement from participants from the beginning of the search process rather than simply during the assessment phase, may be necessary to ensure that the participants receive adequate time to comprehend, reflect on, and analyse the methodologies' practical consequences. Participants also expressed support for the creation of a central decision-making body to carry out horizon scanning of novel healthcare services models and interventions.

For peer review only

Since horizon scanning is a systematic methodology, it may require that the horizon scanner(s) have some level of competency in performing accurate literature searches on the topic of concern. This would imply that prior to the search process, the horizon scanner(s) are aware of the information gaps that need to be filled in accordance with national healthcare priorities and that the horizon scanner(s) may need access to input from national decision makers to shed light on potential implementation challenges such as resource implications, cooperation of stakeholders, ethical and accessibility issues. This could be seen as an essential step for establishing database selection, filtration and prioritisation criteria that would be able to guide the extensive search process and prevent the removal of relevant records of information that meet the stakeholders' needs,[57].

Horizon scanning may be performed by relying solely on secondary sources of data, as demonstrated in this study. However, to increase the probability of attaining "new and emerging" results from a horizon scan, the methodologies may require access directly from policy makers and health care authorities (primary source) to restricted information on models and interventions that are still under development but have not yet been published. Moreover, access to specialised databases of horizon scanning organisations (tertiary source) that can help with search optimisation would be beneficial,[58].

Limitations

Current horizon scanning guidelines from EuroScan and the National Horizon Scanning Centre directed towards pharmaceuticals and health technologies were used in this study,[34, 58]. Even though the guidelines were adapted to fit the study's objectives and ensure validity, these guidelines are generally used to target the early lifecycle of technologies. Health care services, such as integrated care models and interventions, are often already developed and established as practices in a given setting when discussed in the literature or in other sources

For peer review only - http://bmjopen.bmj.com/site/about/guidelines.xhtml

of information. Thus, we found it difficult to scan for "new" initiatives in this context, although they were new to a Norwegian setting.

At the same time, horizon scanning should not be regarded as a systematic literature review,[59]. Signals of "things to come" are detected from manifold information sources in addition to, or even instead of, reviews of scientific literature. Thus, horizon scanning can lack a clear weighting of evidence and should not be misinterpreted to give an exhaustive summary of current evidence. The aim of horizon scanning is rather to inform decision-makers about signs of innovation at an early stage, at which point available information, including information about intervention effect, is limited.

Even though we used guidelines we cannot rule out the possibility that bias was introduced into the scanning's filtration and prioritisation process. During the focus group session, considerations were taken with regards to minimise the moderator's facilitation of conversation, encourage the development of independent viewpoints so that the participants could challenge one another, avoid groupthink, and not be easily influenced by a dominant voice. This was done in addition to sending out the topic guide prior to the session. However, because the participants had limited prior knowledge and potentially a lack of time to establish a good understanding of the horizon scanning methodologies and the nine models and interventions, a limitation of this study could be the reliability of the participants' assessment. With hindsight, the participants should have been given more time in the focus group.

The transferability of the results to other settings may be limited. We carried out a small-scale horizon scanning review with a small sample size, even though each participant had multiple roles in various work settings. This limits the validity of the results through increased bias. In a more comprehensive study, several measures could be taken to improve the validity of the

For peer review only - http://bmjopen.bmj.com/site/about/guidelines.xhtml

results. For example, a Delphi technique could have been used, with an anonymous review, scoring and commenting, before a focus group discussion,[60]. Moreover, involvement of different stakeholder groups, such as policy makers, public and patients, could have been included in the assessment and prioritisation of possible interventions. While the focus group session was in depth, involving diverse stakeholders such as patients and their caregivers as well as increasing the number of participants may have improved the breadth of findings. In addition, conducting multiple focus groups where the models, interventions and horizon scanning methodologies could be discussed and evaluated more comprehensively until no new knowledge is gained from subsequent sessions (saturation), may have strengthened the reliability of the assessments,[60].

CONCLUSION

By using a horizon scanning methodology, new and emerging integrated care models and interventions for the older people with frailty which have the potential to overcome system fragmentation and enhance care coordination have been identified. Furthermore, the horizon scanning process enabled discussion on the need for integrated care and the perceived difficulties of implementing the discussed models and interventions in the Norwegian context. In doing so, horizon scanning may be seen as a valuable tool policy decision makers and healthcare authorities may use for tackling information gaps and creating innovation in service delivery. Further research should look at how the horizon scanning process could be carried out in a real-world environment.

For peer review only - http://bmjopen.bmj.com/site/about/guidelines.xhtml

Abbreviations

PRISMA: The Preferred Reporting Items for Systematic Reviews and Meta-Analyses

COREQ: Consolidated Criteria for Reporting Qualitative Research

Acknowledgements. We thank the participants for their time and efforts.

Contributors All authors contributed to the conception and design of this work. AA was responsible for the acquisition of data under the supervision of EF. AA and EF contributed to analysis and interpretation of data; drafting and revising the paper for intellectual content; final approval of the version to be published and agreed to be accountable for all aspects of the work in ensuring that questions related to the accuracy or integrity of any part of the work are appropriately investigated and resolved.

Funding Financial contribution from the Research Council of Norway through the Norwegian Centre for Health Services Research (NORCHER project number 296114) is acknowledged.

Disclaimer None

Competing interests None declared**.**

Patient consent for publication. Not required**.**

Ethics approval The study was assessed and approved by the Norwegian Centre for Research Data (project number: 948039).

Data sharing statement. All data is available on reasonable request.

Orchid id https://orcid.org/0000-0001-5280-1051

For peer review only - http://bmjopen.bmj.com/site/about/guidelines.xhtml

REFERENCES

1. Clegg A, Young J, Iliffe S, Rikkert MO, Rockwood K. Frailty in elderly people. *Lancet*. 2013;381(9868):752–62.

2. Morley JE, Vellas B, van Kan GA, Anker SD, Bauer JM, Bernabei R, et al. Frailty consensus: a call to action. *J Am Med Dir Assoc*. 2013;14(6):392–7.

3. Rodríguez-Mañas L, Féart C, Mann G, Viña J, Chatterji S, Chodzko-Zajko W, et al. Searching for an operational definition of frailty: a Delphi method based consensus statement: the frailty operative definition-consensus conference project. *J Gerontol A Biol Sci Med Sci.* 2013;68(1):62–7.

4. Mazzola P, Motta L, Picone D, Tabozzi S, Lafortuna C, Bellelli G, et al. FRAILTY AS A DYNAMIC GERIATRIC SYNDROME: ANALYSIS OF THE RISK FACTORS FOR THE ONSET OF FRAILTY USING THE SHARE DATABASE. *Innovation in Aging*. 2018;2:947.

5. Ofori-Asenso R, Chin KL, Mazidi M, Zomer E, Ilomaki J, Zullo AR, et al. Global incidence of frailty and prefrailty among community-dwelling older adults: A systematic review and meta-analysis: A systematic review and meta-analysis. *JAMA Netw Open.* 2019;2(8):e198398.

6. Barnett K, Mercer SW, Norbury M, Watt G, Wyke S, Guthrie B. Epidemiology of multimorbidity and implications for health care, research, and medical education: a cross-sectional study. *Lancet.* 2012;380(9836):37–43.

7. Marengoni A, Angleman S, Melis R, Mangialasche F, Karp A, Garmen A, et al. Aging with multimorbidity: a systematic review of the literature. *Ageing Res Rev.* 2011;10(4):430–9.

For peer review only

8. Dent E, Martin FC, Bergman H, Woo J, Romero-Ortuno R, Walston JD. Management of frailty: opportunities, challenges, and future directions. *Lancet.* 2019;394(10206):1376–86.

9. Gobbens RJJ, Luijkx KG, Wijnen-Sponselee MT, Schols JMGA. Towards an integral conceptual model of frailty. *J Nutr Health Aging*. 2010;14(3):175–81.

10. Hoogendijk EO, Muntinga ME, van Leeuwen KM, van der Horst HE, Deeg DJH, Frijters DHM, et al. Self-perceived met and unmet care needs of frail older adults in primary care. *Arch Gerontol Geriatr.* 2014;58(1):37–42.

11. Vermeiren S, Vella-Azzopardi R, Beckwée D, Habbig A-K, Scafoglieri A, Jansen B, et al. Frailty and the prediction of negative health outcomes: A meta-analysis. *J Am Med Dir Assoc*. 2016;17(12):1163.e1-1163.e17.

12. National Health and Hospital Plan 2020-2023 Norwegian Ministry of Health and Care Services I-1194E

13. World Health Organisation. Integrated care models: an overview [Internet], 2016. Available: https://www.euro.who.int/en/health-topics/Health-systems/health-services-delivery/publications/2016/inte grated-care-models-an-overview-2016

14. Hendry, A, et al. Integrated Care: A Collaborative ADVANTAGE for Frailty. International Journal of Integrated Care, 2018; 18(2): 1, 1–4. DOI: https://doi.org/10.5334/ijic.4156

15. Kodner DL, Spreeuwenberg C. Integrated care: meaning, logic, applications, and implications--a discussion paper. *Int J Integr Care.* 2002;2(4):e12.

16. Apóstolo J, Cooke R, Bobrowicz-Campos E, Santana S, Marcucci M, Cano A, et al. Effectiveness of interventions to prevent pre-frailty and frailty progression in older adults: a systematic review. *JBI Database System Rev Implement Rep*. 2018;16(1):140–232.

For peer review only - http://bmjopen.bmj.com/site/about/guidelines.xhtml

17. Abbasi M, Rolfson D, Khera AS, Dabravolskaj J, Dent E, Xia L. Identification and management of frailty in the primary care setting. *CMAJ*. 2018;190(38):E1134–40.

18. Fairhall N, Kurrle SE, Sherrington C, Lord SR, Lockwood K, John B, et al. Effectiveness of a multifactorial intervention on preventing development of frailty in pre-frail older people: study protocol for a randomised controlled trial. *BMJ Open.* 2015;5(2):e007091.

19. Hines P, Hiu Yu L, Guy RH, Brand A, Papaluca-Amati M. Scanning the horizon: a systematic literature review of methodologies. *BMJ Open.* 2019;9(5):e026764.

20. Amanatidou E, Butter M, Carabias V, Konnola T, Leis M, Saritas O, et al. On concepts and methods in horizon scanning: Lessons from initiating policy dialogues on emerging issues. *Sci Public Policy*. 2012;39(2):208–21.

21. The Institute of Risk Management. Horizon Scanning: A Practitioner's Guide [Internet], 2018. Available: https://www.theirm.org/media/7423/horizon-scanning_final2-1.pdf

22. Urquhart GJ, Saunders P. Wider horizons, wiser choices: horizon scanning for public health protection and improvement. *J Public Health (Oxf).* 2016;fdw039.

23. Miles I, Saritas O. The depth of the horizon: searching, scanning and widening horizons. *Foresight.* 2012;14(6):530–45.

24. Simpson S. EuroScan: A toolkit for the identification and assessment of new and emerging health technologies[Internet], 2014. Available: https://www.euskadi.eus/contenidos/informacion/osteba_formacion_/es_def/adjuntos/EuroScan_Methods_Toolkit_October_2014_FINAL_CC_added.pdf

25. Saunes IS, Karanikolos M, Sagan A. Norway Health system review [Internet], 2020. Available: https://www.who.int/publications/i/item/HiT-22-1-2020

For peer review only - http://bmjopen.bmj.com/site/about/guidelines.xhtml

26. Tjerbo T, Kjekshus L. Coordinating health care: lessons from Norway. *Int J Integr Care*. 2005;5(4):e28.

27. Mur-Veeman I, Hardy B, Steenbergen M, Wistow G. Development of integrated care in England and the Netherlands. *Health Policy*. 2003;65(3):227–41.

28. Ahgren B, Axelsson R. Evaluating integrated health care: a model for measurement. *Int J Integr Care*. 2005;5(3):e01; discussion e03, e09.

29. Curry N, Ham C. The King's Fund: Clinical and service integration: The route to improved outcomes [Internet], 2010. Available: https://www.kingsfund.org.uk/sites/default/files/Clinical-and-service-integration-Natasha-Curry-Chris-Ha m-22-November-2010.pdf

30. Packer C. The National Horizon Scanning Centre (NHSC): Early warning for new and emerging health technologies in England. *Evid-based Healthc Public Health*. 2005;9(6):410–3.

31. Chen CY, Gan P, How CH. Approach to frailty in the elderly in primary care and the community. *Singapore Med J*. 2018;59(5):240–5.

32. European Commission. The 2012 Ageing Report: Economic and Budgetary Projections for the 27 EU Member States (2010-2060) [Internet], 2012. Available: http://ec.europa.eu/economy_finance/

33. Knickman JR, Snell EK. The 2030 problem: caring for aging baby boomers. *Health Serv Res*. 2002;37(4):849–84.

34. World Health Organization. The Global Burden of Disease: 2004 Update [Internet], 2008. Available: https://apps.who.int/iris/bitstream/handle/10665/43942/9789241563710_eng.pdf

For peer review only

35. de Stampa M, Vedel I, Bergman H, Novella J-L, Lechowski L, Ankri J, et al. Opening the black box of clinical collaboration in integrated care models for frail, elderly patients. *Gerontologist.* 2013;53(2):313–25.

36. Pérez LM, Enfedaque-Montes MB, Cesari M, Soto-Bagaria L, Gual N, Burbano MP, et al. A community program of integrated care for frail older adults: +AGIL Barcelona. *J Nutr Health Aging*. 2019;23(8):710–6.

37. Turner G, Clegg A. Best practice guidelines for the management of frailty: a British Geriatrics Society, Age UK and Royal College of General Practitioners report. *Age Ageing*. 2014;43(6):744–7.

38. Kirst M, Im J, Burns T et al – What work in implementation of integrated care programs for older adults with complex needs? A realist review. Int journal for Quality in Health care 2017;29:612-24

39. Looman WM, Huijsman R, Bouwmans-Frijters CAM, Stolk EA, Fabbricotti IN. Cost-effectiveness of the “Walcheren Integrated Care Model” intervention for community-dwelling frail elderly. *Fam Pract*. 2016;33(2):154–60.

40. Beswick AD, Gooberman-Hill R, Smith A, Wylde V, Ebrahim S. Maintaining independence in older people. *Rev Clin Gerontol*. 2010;20(2):128–53.

41. O.Nyumba T, Wilson K, Derrick CJ, Mukherjee N. The use of focus group discussion methodology: Insights from two decades of application in conservation. *Methods Ecol Evol*. 2018;9(1):20–32.

42. Feiring E. Norwegian Centre for Health Service Research (NORCHER)- WP 2: Horizon scanning, assessment and implementation, 2020.

43. Krueger RA, Casey MA. Focus groups: A practical guide for applied research. 5th ed. Thousand Oaks, CA: SAGE Publications; 2014.

For peer review only - http://bmjopen.bmj.com/site/about/guidelines.xhtml

44. Hendry A et al. Integrated care models for managing and preventing frailty: A systematic review for the European Joint Action on Frailty Prevention (ADVANTAGE JA), Translational Medicine 2019, 19(2): 5-10).

45. MacAdam, M. PRISMA: Program of Research to Integrate the Services for the Maintenance of Autonomy. A system-level integration model in Quebec. *Int J Integr Care.* 2015;15(6):e018.

46. Béland F, Bergman H, Lebel P, Clarfield AM, Tousignant P, Contandriopoulos A-P, et al. A system of integrated care for older persons with disabilities in Canada: results from a randomized controlled trial. *J Gerontol A Biol Sci Med Sci*. 2006;61(4):367–73.

47. Looman W, Fabbricotti I, Huijsman R. The cost-effectiveness of an integrated care model for frail elderly: the Walcheren Integrated Care Model. *Int J Integr Care*. 2014;14(6).

48. Hansen JC. Community and in-home models. Am J Nurs. 2008;108(9):72.

49. Counsell SR, Callahan CM, Buttar AB, Clark DO, Frank KI. Geriatric Resources for Assessment and Care of Elders (GRACE): a new model of primary care for low-income seniors: Grace primary care for low-income seniors. *J Am Geriatr Soc.* 2006;54(7):1136–41.

50. FrailSafe - Home [Internet]. Frailsafe-project.eu. [cited 2021 Jul 1]. Available: https://frailsafe-project.eu/

51. van Dijk HM, Cramm JM, Birnie E, Nieboer AP. Effects of an integrated neighborhood approach on older people's (health-related) quality of life and well-being. *BMC Res Notes.* 2016;9(1):450

52. Liotta G, Ussai S, Illario M, O'Caoimh R, Cano A, Holland C, et al. Frailty as the future core business of Public Health: Report of the activities of the A3 Action Group

For peer review only - http://bmjopen.bmj.com/site/about/guidelines.xhtml

of the European innovation partnership on active and healthy ageing (EIP on AHA). *Int J Environ Res Public Health*. 2018;15(12):2843.

53. Healthcare Improvement Scotland. Hospital at Home Guiding principles for service development [Internet], 2020. Available: https://ihub.scot/media/6928/2020205-hospital-at-home-guiding-principles.pdf

54. Duda N, Fleming C, Kirwan B, Roff B, Rich E. Evaluation of the AHRQ Healthcare Horizon Scanning System [Internet], 2016. Available: https://effectivehealthcare.ahrq.gov/sites/default/files/pdf/horizon-scan_research.pdf

55. Hall RH, Lawrence PR, Lorsch JW. Organization and environment: Managing differentiation and integration. *Adm Sci Q*. 1968;13(1):180.

56. Gallego G, Bridges JF, Flynn T, Blauvelt BM. Pcn118 predicting the future impact of emerging technologies on hepatocellular carcinoma (hcc): Measuring stakeholders' preferences with best-worst scaling. *Value Health.* 2011;14(3):A176.

57. United Kingdom government office for science. The Futures Toolkit: Tools for Futures Thinking and Foresight Across UK Government [Internet], 2014. Available: https://www.gov.uk/government/publications/futures-toolkit-for-policy-makers-and-analysts

58. Sun F, Schoelles K. AHRQ HealthCare Horizon Scanning System: A Systematic Review of Methods for Health Care Technology Horizon Scanning [Internet], 2013. Available: https://effectivehealthcare.ahrq.gov/products/horizon-scan/research-2013

59. Cuhls, K. E. Horizon Scanning in Foresight – Why Horizon Scanning is only a part of the game. Futures Foresight Sci. 2020;2:e23 https://doi.org/10.1002/ffo2.23

60. Delphi Technique in Health Sciences: A Map. Niederberger, M , Spranger, J. Frontiers in Public Health 2020 doi: 10.3389/pubh.2020.00457

For peer review only

LEGENDS

Figure 1: Common stages of horizon scanning from the Euroscan Network,[24]. This figure is licensed under the Creative Commons Attribution-NonCommercial-ShareAlike 4.0 International license (CC BT-NC-SA 4.0).

Figure 2: Integrated care models. Adapted from,[27-29].

Figure 3 Horizon scanning process chart.

Figure 4 Overview over the models and interventions detailed by the records,[45-53].

Table 1 Information sources and search terms used for signal detection.

Table 2 Intervention characteristics and considerations used to filter and prioritise models and interventions,[34] ,[39-40].

Table 3 Scoring of models and interventions.

For peer review only

For peer review only - http://bmjopen.bmj.com/site/about/guidelines.xhtml

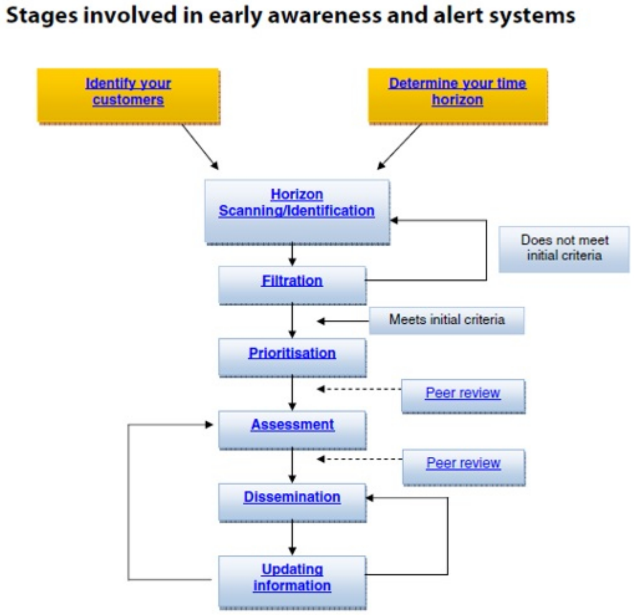

Figure 2 Common stages of horizon scanning from the Euroscan Network,[26]. This figure is licensed under the Creative Commons Attribution-NonCommercial-ShareAlike 4.0 International license (CC BT-NC-SA 4.0).

Figure 1: Common stages of horizon scanning from the Euroscan Network,[24]. This figure is licensed under the Creative Commons Attribution-NonCommercial-ShareAlike 4.0 International license (CC BT-NC-SA 4.0).

68x57mm (300 x 300 DPI)

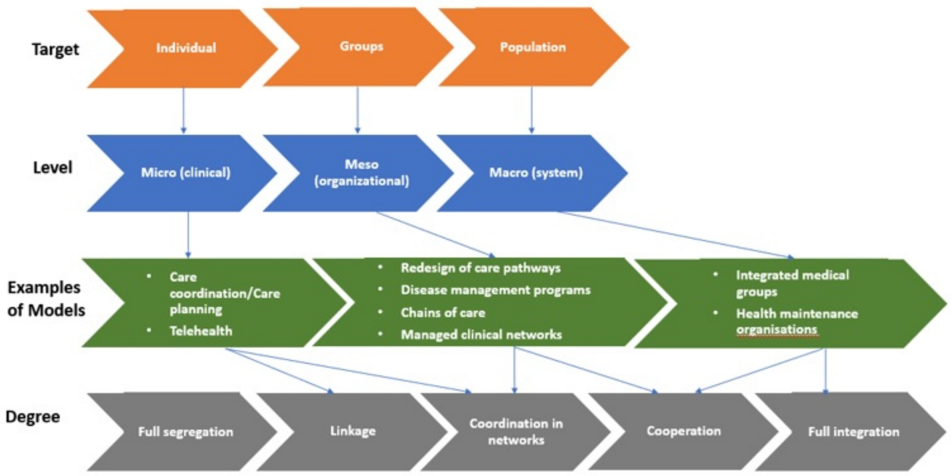

Figure 1 Integrated care models. Adapted from,[19-21].

Figure 2: Integrated care models. Adapted from,[27-29].

66x41mm (300 x 300 DPI)

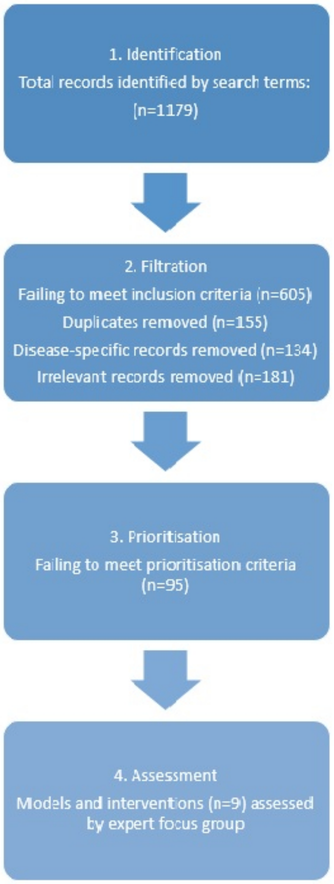

Figure 3 Horizon scanning process chart

Figure 3 Horizon scanning process chart.

66x67mm (300 x 300 DPI)

Title of ICM	PRISMA	SIPA	WICM	PACE/ON LOK	GRACE
	Program of Research to Integrate the Services for the Maintenance of Autonomy	Services intégrés pour les personnes âgées fragiles	Walcheren Integrated Care Model	Program of All-Inclusive Care for the Elderly	Geriatric Resources for Assessment and Care of Elders
**Overview**					
Objectives	Improve continuity and increase the efficacy and efficiency of services	Increased availability of nursing, homemakers, rehabilitation,and social work services would reduce the use and costs of institution-based services	Improve the quality and efficacy of care given to frail elderly living independently	Provide and coordinate cost-effective, comprehensive care for frail adults who want to remain in the community as long as possible but need long term assistance	Provide home-based geriatric specialised coordinated care with focus on both medical and social care (mental health)
General description	Coordination focused integrated service delivery model	Community-based system responsible for also institutional services at no additional cost. Intensive home care, 24 hour on-call availability and rapid team mobilisation	Comprehensive integrated model with focus on the family physician's role as a coordinator of care, proactive detection and assessment of needs for independently living frail elderly	Fully integrated community based model to provide all types of services at one adult day health center	Primary care service program for frail older adults especially those who have low income
Degree of integration: Linkage/Coordination in networks/Cooperation/Full integration	Coordinated provider model: Joint Governing Board for policy, service provision and resource allocation decisions (strategic). Service coordination committee for monitoring of service coordination(tactical) with multidisciplinary team of practitioners (clinical)	Fully integrated provider model: Each SIPA site, 1 program director and administrative support personnel helped to determine its own budget, implementation plan for the patients/services required, partnering agreements and deployment of human resources	Partially integrated provider model: Steering group (umbrella organinsation) consists of representatives from all organisations for the necessary provider network. Family physician is part of the network and refers the patient accordingly.	Fully integrated provider model: providing primary care, specialty care, adult day care, home care, hospital care, nursing-home care, medication oversight, and transportation to medical appointments all at an adult day health care center	Integrated provider model on community medical and social care level; co-ordinated model with acute sector and family physician
Source	(MacAdam, 2015)	(Béland et al., 2006)	(Looman et al., 2014)	(Hansen, 2008)	(Counsell et al., 2006)

Title of Intervention	EuFrailSafe	Integrated neighborhood approach(INA)	Massive Open Online Courses (MOOCs) in Frailty	Hospital at home
**Overview**				
Objectives	Use of advanced technology for frailty assessment, monitoring and developement of personalised frailty health plans to prevent adverse outcomes.	Improve the frail elderly and their self-management skills by connecting with informal networks, available neighbourhood resources before seeking professional help.	Empower the frail and their caregivers by informing them about the aging process in order to increase functional capacity and independence.	To provide individualised care and prevent hospital acquired infections, and functional decline due to stress from transitioning between the departments. To free up hospital beds.
General description	Smart garment (wearable sensor device to monitor medical parameters), indoor localisation application (bluetooth monitoring of movement patterns of the frail at home), games (monitor coodination, decision making skills and reflex)	INA team: Community worker examines the frail elderly needs through interviews, implements individualised care plans involving informal caregivers such as neighbours and volunteers who help to monitor the frail elderly person.	Free online courses providing material as well as a discussion platform for the frail elderly and caregivers to stay informed and be part of an online network.	Emergency access to hospital treatments/diagnostics are provided at home of the frail. Usually consultant-led and care delivered by a multidisciplinary team with the GP.
Degree of integration: Linkage/Coordination in networks/Cooperation/Full integration	Coordination in networks	Coordination in networks+ Coorperation	Coordination in networks	Full integration+ Coordination+ Cooperation
Source	(FrailSafe - Home, 2020)	(van der Heide et al., 2018)	(Liotta et al., 2018)	(Healthcare Improvement Scotland, 2020)

Figure 4 Overview of prioritised system-based integrated care models and community-based interventions,[41-49]

Figure 4 Overview over the models and interventions detailed by the records,[45-53].

84x67mm (300 x 300 DPI)

Preferred Reporting Items for Systematic reviews and Meta-Analyses extension for Scoping Reviews (PRISMA-ScR) Checklist

SECTION	ITEM	PRISMA-ScR CHECKLIST ITEM	REPORTED ON PAGE #
**TITLE**			
Title	1	Identify the report as a scoping review.	
**ABSTRACT**			
Structured summary	2	Provide a structured summary that includes (as applicable): background, objectives, eligibility criteria, sources of evidence, charting methods, results, and conclusions that relate to the review questions and objectives.	
**INTRODUCTION**			
Rationale	3	Describe the rationale for the review in the context of what is already known. Explain why the review questions/objectives lend themselves to a scoping review approach.	
Objectives	4	Provide an explicit statement of the questions and objectives being addressed with reference to their key elements (e.g., population or participants, concepts, and context) or other relevant key elements used to conceptualize the review questions and/or objectives.	
**METHODS**			
Protocol and registration	5	Indicate whether a review protocol exists; state if and where it can be accessed (e.g., a Web address); and if available, provide registration information, including the registration number.	
Eligibility criteria	6	Specify characteristics of the sources of evidence used as eligibility criteria (e.g., years considered, language, and publication status), and provide a rationale.	
Information sources*	7	Describe all information sources in the search (e.g., databases with dates of coverage and contact with authors to identify additional sources), as well as the date the most recent search was executed.	
Search	8	Present the full electronic search strategy for at least 1 database, including any limits used, such that it could be repeated.	
Selection of sources of evidence†	9	State the process for selecting sources of evidence (i.e., screening and eligibility) included in the scoping review.	
Data charting process‡	10	Describe the methods of charting data from the included sources of evidence (e.g., calibrated forms or forms that have been tested by the team before their use, and whether data charting was done independently or in duplicate) and any processes for obtaining and confirming data from investigators.	
Data items	11	List and define all variables for which data were sought and any assumptions and simplifications made.	
Critical appraisal of individual sources of evidence§	12	If done, provide a rationale for conducting a critical appraisal of included sources of evidence; describe the methods used and how this information was used in any data synthesis (if appropriate).	
Synthesis of results	13	Describe the methods of handling and summarizing the data that were charted.	

St. Michael's
Inspired Care. Inspiring Science.

For peer review only - http://bmjopen.bmj.com/site/about/guidelines.xhtml

SECTION	ITEM	PRISMA-ScR CHECKLIST ITEM	REPORTED ON PAGE #
**RESULTS**			
Selection of sources of evidence	14	Give numbers of sources of evidence screened, assessed for eligibility, and included in the review, with reasons for exclusions at each stage, ideally using a flow diagram.	
Characteristics of sources of evidence	15	For each source of evidence, present characteristics for which data were charted and provide the citations.	
Critical appraisal within sources of evidence	16	If done, present data on critical appraisal of included sources of evidence (see item 12).	
Results of individual sources of evidence	17	For each included source of evidence, present the relevant data that were charted that relate to the review questions and objectives.	
Synthesis of results	18	Summarize and/or present the charting results as they relate to the review questions and objectives.	
**DISCUSSION**			
Summary of evidence	19	Summarize the main results (including an overview of concepts, themes, and types of evidence available), link to the review questions and objectives, and consider the relevance to key groups.	
Limitations	20	Discuss the limitations of the scoping review process.	
Conclusions	21	Provide a general interpretation of the results with respect to the review questions and objectives, as well as potential implications and/or next steps.	
**FUNDING**			
Funding	22	Describe sources of funding for the included sources of evidence, as well as sources of funding for the scoping review. Describe the role of the funders of the scoping review.	

JBI = Joanna Briggs Institute; PRISMA-ScR = Preferred Reporting Items for Systematic reviews and Meta-Analyses extension for Scoping Reviews.

* Where *sources of evidence* (see second footnote) are compiled from, such as bibliographic databases, social media platforms, and Web sites.

† A more inclusive/heterogeneous term used to account for the different types of evidence or data sources (e.g., quantitative and/or qualitative research, expert opinion, and policy documents) that may be eligible in a scoping review as opposed to only studies. This is not to be confused with *information sources* (see first footnote).

‡ The frameworks by Arksey and O'Malley (6) and Levac and colleagues (7) and the JBI guidance (4, 5) refer to the process of data extraction in a scoping review as data charting.

§ The process of systematically examining research evidence to assess its validity, results, and relevance before using it to inform a decision. This term is used for items 12 and 19 instead of "risk of bias" (which is more applicable to systematic reviews of interventions) to include and acknowledge the various sources of evidence that may be used in a scoping review (e.g., quantitative and/or qualitative research, expert opinion, and policy document).

From: Tricco AC, Lillie E, Zarin W, O'Brien KK, Colquhoun H, Levac D, et al. PRISMA Extension for Scoping Reviews (PRISMAScR): Checklist and Explanation. Ann Intern Med. 2018;169:467–473. doi: 10.7326/M18-0850.

For peer review only - http://bmjopen.bmj.com/site/about/guidelines.xhtml

COREQ (COnsolidated criteria for REporting Qualitative research) Checklist

A checklist of items that should be included in reports of qualitative research. You must report the page number in your manuscript where you consider each of the items listed in this checklist. If you have not included this information, either revise your manuscript accordingly before submitting or note N/A.

Topic	Item No.	Guide Questions/Description	Reported on Page No.
**Domain 1: Research team and reflexivity**			
Personal characteristics			
Interviewer/facilitator	1	Which author/s conducted the interview or focus group?	
Credentials	2	What were the researcher's credentials? E.g. PhD, MD	
Occupation	3	What was their occupation at the time of the study?	
Gender	4	Was the researcher male or female?	
Experience and training	5	What experience or training did the researcher have?	
Relationship with participants			
Relationship established	6	Was a relationship established prior to study commencement?	
Participant knowledge of the interviewer	7	What did the participants know about the researcher? e.g. personal goals, reasons for doing the research	
Interviewer characteristics	8	What characteristics were reported about the inter viewer/facilitator? e.g. Bias, assumptions, reasons and interests in the research topic	
**Domain 2: Study design**			
Theoretical framework			
Methodological orientation and Theory	9	What methodological orientation was stated to underpin the study? e.g. grounded theory, discourse analysis, ethnography, phenomenology, content analysis	
Participant selection			
Sampling	10	How were participants selected? e.g. purposive, convenience, consecutive, snowball	
Method of approach	11	How were participants approached? e.g. face-to-face, telephone, mail, email	
Sample size	12	How many participants were in the study?	
Non-participation	13	How many people refused to participate or dropped out? Reasons?	
Setting			
Setting of data collection	14	Where was the data collected? e.g. home, clinic, workplace	
Presence of non-participants	15	Was anyone else present besides the participants and researchers?	
Description of sample	16	What are the important characteristics of the sample? e.g. demographic data, date	
Data collection			
Interview guide	17	Were questions, prompts, guides provided by the authors? Was it pilot tested?	
Repeat interviews	18	Were repeat inter views carried out? If yes, how many?	
Audio/visual recording	19	Did the research use audio or visual recording to collect the data?	
Field notes	20	Were field notes made during and/or after the inter view or focus group?	
Duration	21	What was the duration of the inter views or focus group?	
Data saturation	22	Was data saturation discussed?	
Transcripts returned	23	Were transcripts returned to participants for comment and/or	

For peer review only

Topic	Item No.	Guide Questions/Description	Reported on Page No.
		correction?	
**Domain 3: analysis and findings**			
Data analysis			
Number of data coders	24	How many data coders coded the data?	
Description of the coding tree	25	Did authors provide a description of the coding tree?	
Derivation of themes	26	Were themes identified in advance or derived from the data?	
Software	27	What software, if applicable, was used to manage the data?	
Participant checking	28	Did participants provide feedback on the findings?	
Reporting			
Quotations presented	29	Were participant quotations presented to illustrate the themes/findings? Was each quotation identified? e.g. participant number	
Data and findings consistent	30	Was there consistency between the data presented and the findings?	
Clarity of major themes	31	Were major themes clearly presented in the findings?	
Clarity of minor themes	32	Is there a description of diverse cases or discussion of minor themes?	

Developed from: Tong A, Sainsbury P, Craig J. Consolidated criteria for reporting qualitative research (COREQ): a 32-item checklist for interviews and focus groups. *International Journal for Quality in Health Care*. 2007. Volume 19, Number 6: pp. 349 – 357

Once you have completed this checklist, please save a copy and upload it as part of your submission. DO NOT include this checklist as part of the main manuscript document. It must be uploaded as a separate file.

For peer review only

Supplemental material

Supplementary table 1: Backgrounds of participants

Regional health authority	N	Background of participant
Central Norway	4	Senior researcher/ Nurse/ Professor<br>Physiotherapist/ Project manager<br>Advisor<br>Senior Advisor/ Phd-fellow/ Nurse
West	1	Research coordinator/ Nurse specialist (oncology)
South-East	2	Specialist in internal medicine and geriatrics/ Chief physician/ Professor<br>Leader of community services development/ Nurse
Total	7	

For peer review only

Supplementary figure 1: Interview guide

"Horizon scanning of healthcare delivery models within services targeted to the frail elderly"

Focus group agenda

Overall aim of this study

To evaluate if horizon scanning can be used to help decision makers to fill in the knowledge gaps, address issues such as system fragmentation, as well as contribute to innovation of the healthcare delivery services targeted at the frail elderly.

Program

No.	Topic	Focus	Time
1.	Introduction	- Brief description of the study's purpose<br>- Participants' presentation of themselves	1530-1540
2.	Horizon scanning process	- Description of horizon scanning steps<br>- Presentation of results from the scan	1540-1550
3.	System-based models	- Introduction of each model<br>- Discussion<br>- Evaluation	1550-1630
4.	Community-based interventions	- Introduction of each intervention<br>- Discussion<br>- Evaluation	1630-1650
5.	Horizon scanning methodology	- Discussion and evaluation	1650-1700

Interview guide

Do you have any potential conflicts of interest, such as ongoing research or other intellectual / financial interests with organizations related to the models/interventions discussed in this interview?

Yes No

If yes, please describe:

List of innovations

To avoid miscommunication we define new innovations as a possible new way of organizing services, a new mechanism in the service process, changes in the system that increase access to more comprehensive services for frail elderly as well as a new application of existing intervention (s), or other current innovations.

The list is structured after system-based and community-based with an aim to create a better overview for discussion and evaluation. The division is not unambiguous as the integrated care models and community-based interventions do contain overlapping elements.

The innovations placed under "system-based" contain core traits of integrated care models specific for frail elderly on a system/population large scale level.

System-based Innovation	Level of innovation	Comments	Probability of implementation in the next 2-10 years	Comments	Likely impact on frail elderly	Comments
PRISMA						
SIPA						
WICM						
PACE						
GRACE						

Community-based Innovation	Level of innovation	Comments	Probability of implementation in the next 2-10 years	Comments	Likely impact on frail elderly	Comments
EuFrailSafe						
INA						
MOOCs						
Hospital at Home						

Discussion & Evaluation on horizon scanning methodology

What are the current methods you use for making decisions in healthcare service delivery?

What do you think of horizon scanning as a tool for decision making in healthcare service delivery?

What would be the possible strengths and weaknesses of using such a tool?

Any further comments?

iew only

The innovations placed under "community-based" contain traits that allow for the frail to live independently in the community. These have a "door in" approach and are on a more local/community small scale level. This does not mean the community-based are not involved in system level decision making and vice versa.

Discussion & Evaluation on list of innovations

Please reply if you are aware of the mentioned innovations, and, if applicable, leave a comment on the various innovations.

System-based Innovation	Do you know this?	Additional comments
PRISMA	Yes/No	
SIPA	Yes/No	
WICM	Yes/No	
PACE	Yes/No	
GRACE	Yes/No	

Community-based Innovation	Do you know this?	Additional comments
EuFrailSafe	Yes/No	
INA	Yes/No	
MOOCs	Yes/No	
Hospital at Home	Yes/No	

Based on the description and your experience, please rate them on a scale of *low, moderate, and high* accordingly to

i) Level of innovation: degree of novelty, filtration of services from that of common practice.

ii) Probability that the innovation will be further implemented in the next 2-10 years: to see which innovations most likely to be in the horizon of integrated healthcare services for frail elderly. *Things to consider here are resource implications, expected utilisation and availability of the innovation across different geographical areas, actions required before implementation can take place, time, and investment in training of personnel, cooperation of stakeholders and ethical and accessibility issues.*

iii) Likely impact on frail elderly: importance/quality of the innovation. *Things to consider here are the innovation's ability to solve current service issues such as disease-focused treatments, long waiting times, poor exchange of knowledge/collaboration among health workers as a result of not having a shared electronic health record, insufficient staff numbers, lack of guidelines and accountability for care management, absence of professional expertise regarding the patient's health condition, lack of clarity with regards to health personnel's duties and responsibilities as well as a failure in offering updates to patients and their families, along with preparing them for future care transfers.*

System-based Innovation	Level of innovation	Comments	Probability of implementation in the next 2-10 years	Comments	Likely impact on frail elderly	Comments
PRISMA						
SIPA						
WICM						
PACE						
GRACE						

Community-based Innovation	Level of innovation	Comments	Probability of implementation in the next 2-10 years	Comments	Likely impact on frail elderly	Comments
EuFrailSafe						
INA						
MOOCs						
Hospital at Home						

Discussion & Evaluation on horizon scanning methodology

What are the current methods you use for making decisions in healthcare service delivery?

What do you think of horizon scanning as a tool for decision making in healthcare service delivery?

What would be the possible strengths and weaknesses of using such a tool?

Any further comments?

For peer review only

Supplementary table 2: Illustrative quotes from Qualitative assessment

<table>
<tr><td rowspan="2">Shifting away from specialist acute reactive care</td><td>EuFrailSafe</td><td>Hospital at Home</td><td>MOOCs</td><td>WICM</td></tr>
<tr><td>"It is a trend and a need to focus on prevention with the use of technology" Informant 5</td><td>"if it is well-organised within the municipality and we are familiar with the patient's background and medical issues, then it is best and definitely possible to treat them at home." Informant 4</td><td>"This seems to be innovative as it is prevention focused and more customized for the frail people and their caregivers, plus the information is easily accessible" Informant 3</td><td>"I think it would be beneficial if the frail elderly patients were screened early at the doctor's office to avoid hospital admissions" Informant 6</td></tr>
<tr><td rowspan="2">Silos</td><td colspan="2">PRISMA & SIPA</td><td colspan="2">INA</td></tr>
<tr><td colspan="2">"I like that there is a defined team responsible for the patient's care and the focus is on coordination" Informant 2</td><td colspan="2">"A social worker who acts as a coordinator and does assessments at the frail person's home while involving neighbours and volunteers is new and innovative. I have never heard about it. " Informant 7</td></tr>
<tr><td rowspan="2">Service gaps and duplications</td><td>EuFrailSafe</td><td colspan="2">PRISMA & SIPA</td><td>GRACE & PACE</td></tr>
<tr><td>"The virtual platform and use of monitoring devices allows for better clinical follow-up and care" Informant 6"</td><td colspan="2">"The connection between the specialist service and the primary health service is poor, we do not have any communication while in these models there is a team and a platform they use to meet and plan the care for the patient. I think it is a great idea especially for the frail with complex health problems" Informant 7</td><td>"These models seem to have good collaboration routines between the specialist and the primary health service as well as interdisciplinary teams within the primary health service which I feel is important" Informant 1</td></tr>
<tr><td rowspan="2">Competence requirements</td><td colspan="2">MOOCs</td><td colspan="2">WICM</td></tr>
<tr><td colspan="2">" I think it would be useful as you get knowledge about the health condition you are struggling with and support from others" Informant 5</td><td colspan="2">"The idea of a nurse practitioner and family physician teaming up to do geriatric assessments for frailty and early deterioration among elderly is innovative" Informant 1</td></tr>
<tr><td rowspan="2">Greater patient and network involvement</td><td>WICM</td><td>GRACE & PACE</td><td colspan="2">INA</td></tr>
<tr><td>"There is active caregiver support and involvement, that is innovative" Informant 1</td><td>"I believe that in the future with the lack of healthcare personnel and a growing number of elderly, initiatives that involve the network surrounding the frail patient will become essential" Informant 6</td><td colspan="2">"It would be useful and something we would need in the future as there is focus on strengthening social networks in a local environment, where a neighbourhood takes responsibility for the care of the frail." Informant 2</td></tr>
</table>

BMJ Open

Models of integrated care for older people with frailty: A horizon scanning review

Journal:	*BMJ Open*
Manuscript ID	bmjopen-2021-060142.R1
Article Type:	Original research
Date Submitted by the Author:	21-Feb-2022
Complete List of Authors:	A Kjelsnes, Ashwanee; University of Oslo,<br>Feiring, Eli; University of Oslo,
**Primary Subject Heading**:	Health services research
Secondary Subject Heading:	Health policy, Health services research, Public health
Keywords:	Health policy < HEALTH SERVICES ADMINISTRATION & MANAGEMENT, Organisation of health services < HEALTH SERVICES ADMINISTRATION & MANAGEMENT, HEALTH SERVICES ADMINISTRATION & MANAGEMENT

SCHOLARONE™
Manuscripts

I, the Submitting Author has the right to grant and does grant on behalf of all authors of the Work (as defined in the below author licence), an exclusive licence and/or a non-exclusive licence for contributions from authors who are: i) UK Crown employees; ii) where BMJ has agreed a CC-BY licence shall apply, and/or iii) in accordance with the terms applicable for US Federal Government officers or employees acting as part of their official duties; on a worldwide, perpetual, irrevocable, royalty-free basis to BMJ Publishing Group Ltd ("BMJ") its licensees and where the relevant Journal is co-owned by BMJ to the co-owners of the Journal, to publish the Work in this journal and any other BMJ products and to exploit all rights, as set out in our licence.

The Submitting Author accepts and understands that any supply made under these terms is made by BMJ to the Submitting Author unless you are acting as an employee on behalf of your employer or a postgraduate student of an affiliated institution which is paying any applicable article publishing charge ("APC") for Open Access articles. Where the Submitting Author wishes to make the Work available on an Open Access basis (and intends to pay the relevant APC), the terms of reuse of such Open Access shall be governed by a Creative Commons licence – details of these licences and which Creative Commons licence will apply to this Work are set out in our licence referred to above.

Other than as permitted in any relevant BMJ Author's Self Archiving Policies, I confirm this Work has not been accepted for publication elsewhere, is not being considered for publication elsewhere and does not duplicate material already published. I confirm all authors consent to publication of this Work and authorise the granting of this licence.

For peer review only

Models of integrated care for older people with frailty: A horizon scanning review

1 Ashwanee A

Department of Health Management and Health Economics

PO Box 1089 Blindern

0317 Oslo

Norway

Mail: ashwanee.kjelsnes@studmed.uio.no

Orchid id: https://orcid.org/0000-0001-7300-3614

2 Eli Feiring

Department of Health Management and Health Economics

PO Box 1089 Blindern

0317 Oslo

Norway

Orchid id: https://orcid.org/0000-0001-5280-1051

Word count (4379)

For peer review only

ABSTRACT

Objectives Frailty, a multifaceted geriatric condition, is an emerging global health problem. Integrated care models designed to meet the complex needs of the older people with frailty are required. Early identification of innovative models may inform policymakers and other stakeholders of service delivery alternatives they can introduce and locally adapt so as to tackle system fragmentation and lack of coordination. This study used horizon scanning methodologies to systematically search for, prioritise and assess new integrated care models for older people with frailty and investigated experts' views on barriers and facilitators to the adoption of horizon scanning in health services research.

Methods A four-step horizon scanning review was performed. Frailty specific integrated care models and interventions were identified through a review of published literature supplemented with grey literature searches. Results were filtered and prioritised according to pre-set criteria. An expert panel focus group session assessed the prioritised models and interventions on innovativeness, impact and potential for implementation. The experts further evaluated horizon scanning for its perceived fruitfulness in aiding decision-making.

Results Nine integrated care models and interventions at system level (n=5) and community level (n=4) were summarised and assessed by the expert panel (n=7). Test scores were highest for the Walcheren integrated care model (system-based model) and EuFrailSafe (community-based intervention).The participants stated that horizon scanning as a decision making tool, could aid in assessing knowledge gaps, criticising the status quo and developing new insights. Barriers to adoption of horizon scanning on individual, organisational and wider institutional level were also identified.

For peer review only

Conclusion Study findings demonstrated that horizon scanning is a potentially valuable tool in the search for innovative service delivery models. Further studies should evaluate how horizon scanning can be institutionalised and effectively used for serving this purpose.

Strengths and limitations of this study

- The unique contribution of this study is its use of horizon scanning methodologies to identify promising integrated care models and interventions.
- The study's main strength is its systematic method of information mapping, filtration, prioritisation and assessment.
- A limitation is that service models are often already established as practises when reported, thus it is difficult to scan for new interventions in this context.
- A further limitation is that the transferability of results to other setting may be limited.

For peer review only

INTRODUCTION

Frailty, a multifaceted geriatric condition characterised by increased vulnerability to stress incidents due to reductions in reserve and functions in multiple physiological systems, is emerging as a global health problem with significant clinical and public health consequences,[1-4]. It is approximated that 21.3 percent of the world's population will be 60 or older by 2050, where frailty is estimated to affect around one out of every six community-dwelling seniors,[5]. Frailty is associated with a significant increase in comorbid chronic illnesses, functional dependency, disability, healthcare needs and costs,[6-7]. To avoid or delay the progression of frailty to significant functional decline, healthcare designed to meet the complex care requirements is needed,[1, 8-11]. In Norway, as in many other countries, establishing high-quality integrated care for older people with frailty is a political priority,[12]. Integrated care, understood as comprehensive, multi-level and across settings organisation of care, is generally believed to be a solution to the demand for improved care for the multimorbid and long-term care patients,[13]. However, a recent systematic review on integrated care models for managing and preventing frailty concluded that few models were specifically designed to prevent and tackle frailty in the community and at the interface between primary care and secondary care,[14].

The absence of a standardised frailty definition and assessment method coupled with the fact that literature on frailty specific integrated care models and interventions are still in their early stages of development makes it challenging for healthcare decision makers to meet the needs of the older people with frailty,[15-17]. The search for signals of important development in this context can possibly be lessened by horizon scanning, which acts as an information resource that can aid in decisions about the identification of innovative health-care interventions,[18].

Horizon scanning is a systematic approach for detecting early signals of potentially important developments that could impact areas of interest,[19]. It involves a comprehensive review of

For peer review only - http://bmjopen.bmj.com/site/about/guidelines.xhtml

data to bridge knowledge gaps, question assumptions, assess possible threats, challenges and emerging problems, as well as look for opportunities to present new policy alternatives,[20-23]. Signals of "things to come" are detected from manifold information sources in addition to, or even instead of, reviews of scientific literature. These sources include targeted literature searches and input from expert groups, committees, surveys, government bodies, conferences, associations, media and more. Further, experts and other stakeholders with diverse views, experiences, and roles may be brought together to systematically discuss signals as part of the horizon scanning process.

In healthcare, horizon scanning methodologies are commonly used as a health technology assessment (HTA) tool in early awareness and alert (EAA) systems of pharmaceuticals to allow for innovative medicines to enter the market. Less attention has been given to the employment of horizon scanning methodologies in identifying improvements for delivery of healthcare services,[24].

At this backdrop, we wanted to investigate if employing horizon scanning methodologies could be a valuable and viable strategy to identifying novel integrated care initiatives for older people with frailty, in an early phase of adoption. First, we aimed to identify new and emerging integrated care models and interventions that could potentially address system fragmentation issues faced by the older people with frailty and use the opinions of experts to evaluate these models and interventions based on their level of innovation, probability of implementation and impact.

The second aim was to look into experts' opinions on the fruitfulness of employing horizon scanning methodologies in this context, given horizon scanning is still a relatively new tool for identifying innovative healthcare delivery models.

For peer review only - http://bmjopen.bmj.com/site/about/guidelines.xhtml

METHODS

Study design

This study was designed as a small-scale horizon scanning. The PRISMA guidelines were used to report the literature search process as far as possible, and the COREQ guidelines were used to report the findings from the qualitative focus group (supplementary file 1 and 2). The study was notified to and assessed to be in accordance with relevant guidelines by the Norwegian Centre for Research Data (project number: 948039).

Setting

The Norwegian healthcare system is universal, tax-financed, and semi-decentralised,[25]. The responsibility for primary health and social care lies with the municipalities. The central state is responsible for secondary and specialist health care, which is administrated by four Regional Health Authorities (RHAs). The lack of communication between the two tiers of governance contributes to challenges with delivery of integrated care,[26]. Although a Coordination Reform (2012) established mandatory network governance to improve coordination between primary and specialist care, integrated care involving different levels is hindered by lack of formalised coordination and cooperation between the municipalities and the hospitals,[12].

Horizon scanning to identify novel integrated care models

Horizon scanning generally follows a six-step approach of signal detection, filtration, prioritisation, assessment, and dissemination and updating information (figure 1). The first step often includes mapping signals of innovation with the use of literature reviews, including

For peer review only - http://bmjopen.bmj.com/site/about/guidelines.xhtml

reviews of grey literature and reports retrieved from governmental bodies, conferences, meetings, forums, observatories, and other organisations. Pre-set filtration and prioritisation criteria are used to discard irrelevant signals. Assessment methods include participation of experts, users and policymakers, and peer reviews. The results of the horizon scanning are then disseminated and evaluated,[24].

A horizon scanning may be carried out at the beginning of a broader foresight process, aiming to address the full cycle of policy on “complex futures” and involving a range of stakeholders, long-term considerations and different scenarios. It may, however, also be a stand-alone approach for identifying “things to come”. In the present study, the horizon scanning process carried out followed the first four steps of the EuroScan methods toolkit for early awareness and alert systems (EAAS),[24]. We conducted a focus group session to obtain thoughts on integrated care needs for older people with frailty, as well as opinions on the models and interventions identified in the literature and perspectives on horizon scanning methodologies and its potential consequences.

We followed a multifaceted definition of “integrated care” in this study. Integrated care models can be organised according to target group, level and degree (figure 2). Thus, we kept a broad understanding of integrated care as an organisational coordination mechanism that can be understood as to providing a cohesive and continuum of care that is personalised to the patient's condition,[27-29].

Figure 1

Figure 2

Literature search strategy: Identification, filtration and prioritisation of records

For peer review only - http://bmjopen.bmj.com/site/about/guidelines.xhtml

Search strategy

Reviews of published literature and grey literature were performed to trace new and emerging integrated care models and interventions, targeted at the older people with frailty, which had the potential in addressing system fragmentation issues. Databases and governmental bodies were searched using pre-specified search terms to identify research papers, proceedings of conferences and workshops, policy papers and reports (Table 1). Only records published in English or Norwegian were included. The final search took place from 01.11.2020 to 01.02.2021.

Information sources	Search terms
Online databases<br>• Pubmed (384)<br>• Cochrane Library (19)<br>• Evidence-based medical reviews (24)<br>• Embase (349)<br>• Oria UiO (50)<br>• JStor (92)<br>• Medline Ovid (27)<br>• Web of Science (41)<br>• Scopus (104)	• Frail elderly<br>• Integrated care model<br>• Multidisciplinary<br>• Aged care<br>• Service delivery model<br>• Older people<br>• Geriatric<br>• >65<br>• Health sciences<br>• Political sciences<br>• Public health<br>• Public policy and administration
Governmental reports and conferences	

For peer review only

• Norwegian institute of public health (20)<br>• The Norwegian Directorate of Health (29)<br>• Ministry of Health and Care Services (10)<br>• Norwegian National Advisory Unit on Ageing and Health (28)<br>• The innovation conference: the outward-looking hospital (1)<br>• Frailty among the elderly conference (1)	• Health policy

Table 1 Information sources and search terms used for signal detection

Inclusion and exclusion criteria

Findings were filtered by scanning each record's abstract, title and keywords based on a set of inclusion and exclusion criteria, which were adapted from EuroScan,[24] and the National Horizon Scanning Centre (NHSC) guidelines for horizon scanning,[30] as well as from previous literature. Records that dealt with the adoption, execution, or assessment of initiatives focused on the concept of patient-centred integration: "funding, administrative, organisational, service delivery, and clinical levels required to promote interaction, coordination, and cooperation in and between the cure and care sectors were included,[15].

Records focused solely on integrated care, multidisciplinary team and frailty without describing any intervention and/or model, as well as those not specifically focused on the older people with frailty, were excluded. Disease-specific publications were removed because frailty is considered a multi-faceted and dynamic disease,[31-38].

A range of integrated care models and interventions were identified in the material. The different initiatives have been developed to be applied at different key points in the frailty

For peer review only - http://bmjopen.bmj.com/site/about/guidelines.xhtml

For peer review only

care pathway,[39] such as preventive education, enablement and care and support at home, assessment at management in primary care, geriatric assessment in hospital and intermediate care services,[39]. We chose to group the remaining records into two groups with an aim to create a better overview for discussion and evaluation. First, we identified initiatives developed to give comprehensive integrated chronic care and we categorised these models as " system-level integrated care models". Second, we categorised more discrete interventions that provide specific components of integrated care as "community-based interventions".. The grouping was not unambiguous as the integrated care models and community-based interventions do contain overlapping elements. We included records that described models that had some or all of the characteristics illustrated in Table 2.

Prioritisation of models and interventions prior to focus group assessment

Prior to focus group assessment, we did a criteria-informed qualitative prioritisation of the system-based models and community-based interventions (Table 2). The aim of the prioritisation was to identify models and interventions not yet implemented or tested in a Norwegian setting, which we considered to have the potential to address system fragmentation issues.

System-level integrated care models for older people with frailty	Community-based interventions for older people with frailty.	Prioritisation criteria
• Centralised point of entry<br>• Geriatric evaluations<br>• Case management	• Local or community level-based interventions<br>• Living-at-home	• Potential care outcomes<br>• Potential cost-effectiveness

For peer review only - http://bmjopen.bmj.com/site/about/guidelines.xhtml

• Multidisciplinary teams<br>• Multidisciplinary guidelines and meetings<br>• Digitalised patient files<br>• Network framework	• Measures described to promote independence	• Expected resource utilisation<br>• Expected reorganisation of services<br>• Applicability<br>• Novelty<br>• Forward thinking

Table 2 Intervention characteristics and considerations used to filter and prioritise models and interventions,[34] ,[40-41].

Focus group: Assessment of records

Participants and recruitment

The focus group's goal was to discuss and assess the literature review's findings. Purposive sampling was used to recruit participants that had a variety of roles and educational backgrounds as well as knowledge of services provided to the older people with frailty,[42]. The research team approached the Norwegian National Advisory Unit on Ageing and Health and was set in contact with potential participants that were subsequently invited to the study. The invitees further provided potential participants (snowball sampling). Eleven persons were invited to participate.

Data collection

The focus group was conducted on 07.04.2021 via Zoom by AA. Consent forms were signed and collected prior to the focus group.

For peer review only - http://bmjopen.bmj.com/site/about/guidelines.xhtml

Prior to the focus group discussion, the participants were emailed information on the horizon scanning process conducted, tables of the identified models and interventions, as well as the semi-structured topic guide (supplemental material figure 1). They were asked to score and evaluate the different models independently, but we did not collect their evaluations before the focus group took place. This was a pragmatic choice given the study's time- and resource limits.

The focus group session was divided into three sections. The first section presented a summary of the horizon scanning process as well as the key features of each model and intervention. This was done to clear up any misunderstandings or questions they had about the models and horizon scanning process. The models and interventions were organised and presented in accordance with the various forms of integration, with the aim of demonstrating how they provided complex integrated care to the frail in a clear and understandable manner. To avoid miscommunication among the participants, "innovations" were defined as i) as a possible new way of organising services, ii) a new mechanism in the service process, iii) changes in the system that increase access to more comprehensive services for older people with frailty, iv) a new application of existing intervention(s), or other current innovations,[43].

The second section focused on assessment of the models and interventions where the participants were asked to collectively discuss, reflect and rate each model and intervention on a scale from low to high, on the following equally-weighted aspects; level of innovation, probability for implementation in the next 2-10 years, and potential impact on the older people with frailty. Further details of what these three aspects meant were also included in the interview guide (supplemental material figure 1). In the third section, participants were finally asked to offer their thoughts on horizon scanning, its prospective implications and potential for use as a decision-making tool.

For peer review only - http://bmjopen.bmj.com/site/about/guidelines.xhtml

The focus group session lasted two hours. Discussions were recorded on a password-protected computer connected to a university server. The transcription was done through coding to protect the anonymity of the participants.

Data analysis

Organisation and analysis of data collected from the focus group discussion followed the continuum of data analysis framework,[44]. Data were transcribed and organised according to the topic guide ensuring that both positive and negative comments with regards to each model and intervention evaluated against the three criteria, were included. The descriptive statements were then indexed, arranged, compared, analysed and rearranged to create categories for both quantitative and qualitative results. Data used as illustrative purposes were translated from Norwegian to English by the authors.

Patient or public involvement

Patients and public were not involved in any part of our research.

RESULTS

Identification, filtration and prioritisation

There were 1179 records identified through the initial database searches and grey literature, of which 605 were removed due to failing to meet the inclusion criteria at the filtration stage. One hundred and fifty-five duplicates and 134 disease-specific records were excluded, and 181 records were thereafter removed after reading through the full text for relevance. At the prioritisation stage, one hundred and four records were read and evaluated according to the prioritisation criteria. Nine records were included in this study after prioritisation (figure 3).

For peer review only - http://bmjopen.bmj.com/site/about/guidelines.xhtml

Five system-based models and four community-based interventions,[45-53] were prioritised to be assessed in the focus group (figure 4), as described in the Methods section.

Figure 3

Figure 4

Evaluation

Participants

Eleven persons were invited to participate in the focus group; four declined the invitation due to other work commitments. The seven participants that took part were experienced healthcare professionals with various educational backgrounds and had multiple roles in academia, specialist and primary care. They resided in different parts of the country (supplemental material table 1).

Quantitative scores

The participants discussed and then agreed on a score for each system- level integrated care models and community-based interventions together on the three aspects: innovation, implementation, and impact on a low, moderate, and high scale. The scores are stated below in Table 3.

System-based integrated care model	Level of innovation	Probability of implementation in the next 2-10 years	Likely impact on frail elderly
PRISMA	L	L/M	M
SIPA	L	L/M	M
WICM	L/M	H	M/H
PACE	L/M	L	M
GRACE	L/M	L	M

For peer review only - http://bmjopen.bmj.com/site/about/guidelines.xhtml

Community-based intervention	Level of innovation	Probability of implementation in the next 2-10 years	Likely impact on frail elderly
EuFrailSafe	H	H	H
INA	H	M	M/H
MOOCs	M/H	M/H	M
Hospital at Home	M	M/H	M/H

Table 3 Scoring of models and interventions

The Walcheren Integrated Care Model (WICM) had the overall highest scores among the system-based integrated care models. It received low to moderate scores of innovation, high probability of implementation as well as moderate to high impact on older people with frailty which referred to the model's ability to solve current care delivery issues such as lack guidelines and accountability for care management. None of the system-based integrated care models were regarded as particularly innovative and all had moderate impact on the older people with frailty. In terms of the community-based interventions, EuFrailSafe had the overall highest scores with high scores on all three categories. None of the community-based interventions scored low in any category.

Qualitative assessment

The quantitative scores were further substantiated by qualitative assessments where the participants commented on how the five system-based integrated care models and four community-based interventions could help solve system fragmentation issues (supplemental material table 2). The participants stated how innovative service delivery approaches targeted at the older people with frailty should involve these themes, (i) an assigned frail coordinator, (ii) integrated patient information systems, (iii) multidisciplinary teamwork, (iv) competency within frailty, (v) patient and network empowerment as well as a (vi) shift from specialist acute reactive care to primary preventative, proactive care.

For peer review only - http://bmjopen.bmj.com/site/about/guidelines.xhtml

For peer review only

For example, the system-based WICM model was seen to be favourable due to its focus on community care, teamwork, and caregiver involvement. However, despite the consensus among participants that certain traits of *system-based integrated care models* were considered vital for delivering holistic care(i.e., caregiver support in PACE and GRACE, proactive detection for frailty in WICM and a frailty coordinator in PRISMA and SIPA), there was uncertainty about how they would be adapted and applied in the Norwegian context.

The participants viewed *community-based interventions* focusing on welfare technology (EuFrailSafe), active social network participation (INA), comprehensive home care services (Hospital at Home), and frailty education (MOOCs) as both in line with frailty care needs and trends as well as easily adaptable to the Norwegian environment. The use of technological devices, such as described in the EuFrailSafe model, was highlighted as innovative.

Horizon scanning as a decision-making tool

Horizon scanning, according to the participants, could be a valuable decision-making tool as it involved assessing knowledge gaps, criticising the status quo, developing new insights on the topic of concern, and networking with experts prior to the implementation of measures.

> *It is a method for gaining more knowledge and translating it into practice with expert assessments. It can be a way to collaborate with other knowledge communities, once you have identified an information gap* Participant 2.

In addition, the participants emphasised that the method would necessitate expertise and should be carried out by policymakers to shed light on possible implementational challenges.

> *The method requires good systematic literature search. That is the foundation of the process. Not everyone can do that. The filtering and prioritisation criteria are choices*

For peer review only

one needs to make and if unsure, the process can give the wrong results. It is a subject of its own, so it has to be done at a higher organisational level Participant 5.

The participants expressed that the results of the horizon scan were challenging to comprehend and evaluate.

These models are complex, and it is difficult to get an overall understanding of them Participant 4.

DISCUSSION

In line with the study's objectives, the small-scale horizon scan conducted in this study identified novel integrated care models and interventions, the majority of which were regarded by the participants as innovative, had the potential to impact the older people with frailty and were appropriate to some degree, for implementation in the Norwegian healthcare system. Additionally, the discussion of models and interventions were able to give the participants insight into needs and trends of integrated care as well as alternative solutions to address information gaps, system fragmentation and current service innovation.

However, participants raised some concerns about the potential adaptability and applicability of the system-based integrated care models to a Norwegian context. This finding is not surprising. Studies of integrated care models suggest that the higher the level of integration specified by the design, the higher the level of differentiation,[54-55]. In Norway, integrated care involving different decision-making levels is hindered by lack of formalised coordination and cooperation between hospitals and municipalities,[21]. Thus, in this setting, the various components of integration present in the system-based models necessitate large-scale changes in legal and financial regulations, as well as organisational reorganisation and thus, government support for implementation would be required.

For peer review only

On the other hand, the participants gave high scores to the more discrete interventions focusing on specific components of integrated care at the community level. As many of the participants held municipal-level positions, it may have been easier for them to envision how these interventions could be implemented without requiring major legislative changes.

In this study, it was assumed that criteria such as potential for impact, innovativeness and implementation are equally weighted. It is important to note that the scores can be changed as policymakers and healthcare authorities may weigh these criteria differently based on the country's healthcare goals,[56].

According to the participants, horizon scanning was deemed a beneficial tool to employ as it entailed assessing knowledge gaps, questioning the status quo, getting new perspectives on approaching the topic of concern, and networking with other experts prior to implementing interventions. However, there were varying opinions on the process's practical application. This uncertainty may be due to the study's participants having little to no prior knowledge of horizon scanning and its use in decision making. Involvement from participants from the beginning of the search process rather than simply during the assessment phase, may be necessary to ensure that the participants receive adequate time to comprehend, reflect on, and analyse the methodologies' practical consequences. Participants also expressed support for the creation of a central decision-making body to carry out horizon scanning of novel healthcare services models and interventions.

Since horizon scanning is a systematic methodology, it may require that the horizon scanner(s) have some level of competency in performing accurate literature searches on the topic of concern. This would imply that prior to the search process, the horizon scanner(s) are aware of the information gaps that need to be filled in accordance with national healthcare priorities and that the horizon scanner(s) may need access to input from national decision

For peer review only

makers to shed light on potential implementation challenges such as resource implications, cooperation of stakeholders, ethical and accessibility issues. This could be seen as an essential step for establishing database selection, filtration and prioritisation criteria that would be able to guide the extensive search process and prevent the removal of relevant records of information that meet the stakeholders' needs,[57].

Horizon scanning may be performed by relying solely on secondary sources of data, as demonstrated in this study. However, to increase the probability of attaining "new and emerging" results from a horizon scan, the methodologies may require access directly from policy makers and health care authorities (primary source) to restricted information on models and interventions that are still under development but have not yet been published. Moreover, access to specialised databases of horizon scanning organisations (tertiary source) that can help with search optimisation would be beneficial,[58].

Limitations

Current horizon scanning guidelines from EuroScan and the National Horizon Scanning Centre directed towards pharmaceuticals and health technologies were used in this study,[34, 58]. Even though the guidelines were adapted to fit the study's objectives and ensure validity, these guidelines are generally used to target the early lifecycle of technologies. Health care services, such as integrated care models and interventions, are often already developed and established as practices in a given setting when discussed in the literature or in other sources of information. Thus, we found it difficult to scan for "new" initiatives in this context, although they were new to a Norwegian setting.

At the same time, horizon scanning should not be regarded as a systematic literature review,[59]. Signals of "things to come" are detected from manifold information sources in addition to, or even instead of, reviews of scientific literature. Thus, horizon scanning can

For peer review only - http://bmjopen.bmj.com/site/about/guidelines.xhtml

lack a clear weighting of evidence and should not be misinterpreted to give an exhaustive summary of current evidence. The aim of horizon scanning is rather to inform decision-makers about signs of innovation at an early stage, at which point available information, including information about intervention effect, is limited.

Even though we used guidelines we cannot rule out the possibility that bias was introduced into the scanning's filtration and prioritisation process. During the focus group session, considerations were taken with regards to minimise the moderator's facilitation of conversation, encourage the development of independent viewpoints so that the participants could challenge one another, avoid groupthink, and not be easily influenced by a dominant voice. This was done in addition to sending out the topic guide prior to the session. However, because the participants had limited prior knowledge and potentially a lack of time to establish a good understanding of the horizon scanning methodologies and the nine models and interventions, a limitation of this study could be the reliability of the participants' assessment. With hindsight, the participants should have been given more time in the focus group.

The transferability of the results to other settings may be limited. We carried out a small-scale horizon scanning review with a small sample size, even though each participant had multiple roles in various work settings. This limits the validity of the results through increased bias. In a more comprehensive study, several measures could be taken to improve the validity of the results. For example, a Delphi technique could have been used, with an anonymous review, scoring and commenting, before a focus group discussion,[60]. Moreover, involvement of different stakeholder groups, such as policy makers, public and patients, could have been included in the assessment and prioritisation of possible interventions. While the focus group session was in depth, involving diverse stakeholders such as patients and their caregivers as

For peer review only - http://bmjopen.bmj.com/site/about/guidelines.xhtml

well as increasing the number of participants may have improved the breadth of findings. In addition, conducting multiple focus groups where the models, interventions and horizon scanning methodologies could be discussed and evaluated more comprehensively until no new knowledge is gained from subsequent sessions (saturation), may have strengthened the reliability of the assessments,[60].

CONCLUSION

By using a horizon scanning methodology, new and emerging integrated care models and interventions for the older people with frailty which have the potential to overcome system fragmentation and enhance care coordination have been identified. Furthermore, the horizon scanning process enabled discussion on the need for integrated care and the perceived difficulties of implementing the discussed models and interventions in the Norwegian context. In doing so, horizon scanning may be seen as a valuable tool policy decision makers and healthcare authorities may use for tackling information gaps and creating innovation in service delivery. Further research should look at how the horizon scanning process could be carried out in a real-world environment.

For peer review only

Abbreviations

PRISMA: The Preferred Reporting Items for Systematic Reviews and Meta-Analyses

COREQ: Consolidated Criteria for Reporting Qualitative Research

For peer review only - http://bmjopen.bmj.com/site/about/guidelines.xhtml

Acknowledgements. We thank the participants for their time and efforts.

Contributors All authors contributed to the conception and design of this work. AA was responsible for the acquisition of data under the supervision of EF. AA and EF contributed to analysis and interpretation of data; drafting and revising the paper for intellectual content; final approval of the version to be published and agreed to be accountable for all aspects of the work in ensuring that questions related to the accuracy or integrity of any part of the work are appropriately investigated and resolved.

Funding Financial contribution from the Research Council of Norway through the Norwegian Centre for Health Services Research (NORCHER project number 296114) is acknowledged.

Disclaimer None

Competing interests None declared.

Patient consent for publication. Not required.

Ethics approval The study was assessed and approved by the Norwegian Centre for Research Data (project number: 948039).

Data sharing statement. All data is available on reasonable request.

Orchid id https://orcid.org/0000-0001-5280-1051

For peer review only

REFERENCES

1. Clegg A, Young J, Iliffe S, Rikkert MO, Rockwood K. Frailty in elderly people. *Lancet*. 2013;381(9868):752–62.

2. Morley JE, Vellas B, van Kan GA, Anker SD, Bauer JM, Bernabei R, et al. Frailty consensus: a call to action. *J Am Med Dir Assoc*. 2013;14(6):392–7.

For peer review only - http://bmjopen.bmj.com/site/about/guidelines.xhtml

3. Rodríguez-Mañas L, Féart C, Mann G, Viña J, Chatterji S, Chodzko-Zajko W, et al. Searching for an operational definition of frailty: a Delphi method based consensus statement: the frailty operative definition-consensus conference project. *J Gerontol A Biol Sci Med Sci.* 2013;68(1):62–7.

4. Mazzola P, Motta L, Picone D, Tabozzi S, Lafortuna C, Bellelli G, et al. FRAILTY AS A DYNAMIC GERIATRIC SYNDROME: ANALYSIS OF THE RISK FACTORS FOR THE ONSET OF FRAILTY USING THE SHARE DATABASE. *Innovation in Aging*. 2018;2:947.

5. Ofori-Asenso R, Chin KL, Mazidi M, Zomer E, Ilomaki J, Zullo AR, et al. Global incidence of frailty and prefrailty among community-dwelling older adults: A systematic review and meta-analysis: A systematic review and meta-analysis. *JAMA Netw Open.* 2019;2(8):e198398.

6. Barnett K, Mercer SW, Norbury M, Watt G, Wyke S, Guthrie B. Epidemiology of multimorbidity and implications for health care, research, and medical education: a cross-sectional study. *Lancet.* 2012;380(9836):37–43.

7. Marengoni A, Angleman S, Melis R, Mangialasche F, Karp A, Garmen A, et al. Aging with multimorbidity: a systematic review of the literature. *Ageing Res Rev.* 2011;10(4):430–9.

8. Dent E, Martin FC, Bergman H, Woo J, Romero-Ortuno R, Walston JD. Management of frailty: opportunities, challenges, and future directions. *Lancet.* 2019;394(10206):1376–86.

9. Gobbens RJJ, Luijkx KG, Wijnen-Sponselee MT, Schols JMGA. Towards an integral conceptual model of frailty. *J Nutr Health Aging*. 2010;14(3):175–81.

For peer review only - http://bmjopen.bmj.com/site/about/guidelines.xhtml

10. Hoogendijk EO, Muntinga ME, van Leeuwen KM, van der Horst HE, Deeg DJH, Frijters DHM, et al. Self-perceived met and unmet care needs of frail older adults in primary care. *Arch Gerontol Geriatr.* 2014;58(1):37–42.

11. Vermeiren S, Vella-Azzopardi R, Beckwée D, Habbig A-K, Scafoglieri A, Jansen B, et al. Frailty and the prediction of negative health outcomes: A meta-analysis. *J Am Med Dir Assoc*. 2016;17(12):1163.e1-1163.e17.

12. National Health and Hospital Plan 2020-2023 Norwegian Ministry of Health and Care Services I-1194E

13. World Health Organisation. Integrated care models: an overview [Internet], 2016. Available: https://www.euro.who.int/en/health-topics/Health-systems/health-services-delivery/publications/2016/inte grated-care-models-an-overview-2016

14. Hendry, A, et al. Integrated Care: A Collaborative ADVANTAGE for Frailty. International Journal of Integrated Care, 2018; 18(2): 1, 1–4. DOI: https://doi.org/10.5334/ijic.4156

15. Kodner DL, Spreeuwenberg C. Integrated care: meaning, logic, applications, and implications--a discussion paper. *Int J Integr Care.* 2002;2(4):e12.

16. Apóstolo J, Cooke R, Bobrowicz-Campos E, Santana S, Marcucci M, Cano A, et al. Effectiveness of interventions to prevent pre-frailty and frailty progression in older adults: a systematic review. *JBI Database System Rev Implement Rep*. 2018;16(1):140–232.

17. Abbasi M, Rolfson D, Khera AS, Dabravolskaj J, Dent E, Xia L. Identification and management of frailty in the primary care setting. *CMAJ.* 2018;190(38):E1134–40.

18. Fairhall N, Kurrle SE, Sherrington C, Lord SR, Lockwood K, John B, et al. Effectiveness of a multifactorial intervention on preventing development of frailty in

For peer review only

pre-frail older people: study protocol for a randomised controlled trial. *BMJ Open.* 2015;5(2):e007091.

19. Hines P, Hiu Yu L, Guy RH, Brand A, Papaluca-Amati M. Scanning the horizon: a systematic literature review of methodologies. *BMJ Open.* 2019;9(5):e026764.

20. Amanatidou E, Butter M, Carabias V, Konnola T, Leis M, Saritas O, et al. On concepts and methods in horizon scanning: Lessons from initiating policy dialogues on emerging issues. *Sci Public Policy*. 2012;39(2):208–21.

21. The Institute of Risk Management. Horizon Scanning: A Practitioner's Guide [Internet], 2018. Available: https://www.theirm.org/media/7423/horizon-scanning_final2-1.pdf

22. Urquhart GJ, Saunders P. Wider horizons, wiser choices: horizon scanning for public health protection and improvement. *J Public Health (Oxf).* 2016;fdw039.

23. Miles I, Saritas O. The depth of the horizon: searching, scanning and widening horizons. *Foresight.* 2012;14(6):530–45.

24. Simpson S. EuroScan: A toolkit for the identification and assessment of new and emerging health technologies[Internet], 2014. Available: https://www.euskadi.eus/contenidos/informacion/osteba_formacion_/es_def/adjuntos/EuroScan_Methods_Toolkit_October_2014_FINAL_CC_added.pdf

25. Saunes IS, Karanikolos M, Sagan A. Norway Health system review [Internet], 2020. Available: https://www.who.int/publications/i/item/HiT-22-1-2020

26. Tjerbo T, Kjekshus L. Coordinating health care: lessons from Norway. *Int J Integr Care*. 2005;5(4):e28.

27. Mur-Veeman I, Hardy B, Steenbergen M, Wistow G. Development of integrated care in England and the Netherlands. *Health Policy.* 2003;65(3):227–41.

For peer review only - http://bmjopen.bmj.com/site/about/guidelines.xhtml

28. Ahgren B, Axelsson R. Evaluating integrated health care: a model for measurement. *Int J Integr Care*. 2005;5(3):e01; discussion e03, e09.

29. Curry N, Ham C. The King's Fund: Clinical and service integration: The route to improved outcomes [Internet], 2010. Available: https://www.kingsfund.org.uk/sites/default/files/Clinical-and-service-integration-Natasha-Curry-Chris-Ha m-22-November-2010.pdf

30. Packer C. The National Horizon Scanning Centre (NHSC): Early warning for new and emerging health technologies in England. *Evid-based Healthc Public Health*. 2005;9(6):410–3.

31. Chen CY, Gan P, How CH. Approach to frailty in the elderly in primary care and the community. *Singapore Med J*. 2018;59(5):240–5.

32. European Commission. The 2012 Ageing Report: Economic and Budgetary Projections for the 27 EU Member States (2010-2060) [Internet], 2012. Available: http://ec.europa.eu/economy_finance/

33. Knickman JR, Snell EK. The 2030 problem: caring for aging baby boomers. *Health Serv Res*. 2002;37(4):849–84.

34. World Health Organization. The Global Burden of Disease: 2004 Update [Internet], 2008. Available: https://apps.who.int/iris/bitstream/handle/10665/43942/9789241563710_eng.pdf

35. de Stampa M, Vedel I, Bergman H, Novella J-L, Lechowski L, Ankri J, et al. Opening the black box of clinical collaboration in integrated care models for frail, elderly patients. *Gerontologist*. 2013;53(2):313–25.

36. Pérez LM, Enfedaque-Montes MB, Cesari M, Soto-Bagaria L, Gual N, Burbano MP, et al. A community program of integrated care for frail older adults: +AGIL Barcelona. *J Nutr Health Aging*. 2019;23(8):710–6.

For peer review only - http://bmjopen.bmj.com/site/about/guidelines.xhtml

37. Turner G, Clegg A. Best practice guidelines for the management of frailty: a British Geriatrics Society, Age UK and Royal College of General Practitioners report. *Age Ageing*. 2014;43(6):744–7.

38. Kirst M, Im J, Burns T et al – What work in implementation of integrated care programs for older adults with complex needs? A realist review. Int journal for Quality in Health care 2017;29:612-24

39Hendry A et al. Integrated care models for managing and preventing frailty: A systematic review for the European Joint Action on Frailty Prevention (ADVANTAGE JA), Translational Medicine 2019, 19(2): 5-10).

40. Looman WM, Huijsman R, Bouwmans-Frijters CAM, Stolk EA, Fabbricotti IN. Cost-effectiveness of the "Walcheren Integrated Care Model" intervention for community-dwelling frail elderly. *Fam Pract*. 2016;33(2):154–60.

41. Beswick AD, Gooberman-Hill R, Smith A, Wylde V, Ebrahim S. Maintaining independence in older people. *Rev Clin Gerontol*. 2010;20(2):128–53.

42. O.Nyumba T, Wilson K, Derrick CJ, Mukherjee N. The use of focus group discussion methodology: Insights from two decades of application in conservation. *Methods Ecol Evol*. 2018;9(1):20–32.

43. Feiring E. Norwegian Centre for Health Service Research (NORCHER)- WP 2: Horizon scanning, assessment and implementation, 2020.

44. Krueger RA, Casey MA. Focus groups: A practical guide for applied research. 5th ed. Thousand Oaks, CA: SAGE Publications; 2014.

45. MacAdam, M. PRISMA: Program of Research to Integrate the Services for the Maintenance of Autonomy. A system-level integration model in Quebec. *Int J Integr Care*. 2015;15(6):e018.

For peer review only

46. Béland F, Bergman H, Lebel P, Clarfield AM, Tousignant P, Contandriopoulos A-P, et al. A system of integrated care for older persons with disabilities in Canada: results from a randomized controlled trial. *J Gerontol A Biol Sci Med Sci*. 2006;61(4):367–73.

47. Looman W, Fabbricotti I, Huijsman R. The cost-effectiveness of an integrated care model for frail elderly: the Walcheren Integrated Care Model. *Int J Integr Care*. 2014;14(6).

48. Hansen JC. Community and in-home models. Am J Nurs. 2008;108(9):72.

49. Counsell SR, Callahan CM, Buttar AB, Clark DO, Frank KI. Geriatric Resources for Assessment and Care of Elders (GRACE): a new model of primary care for low-income seniors: Grace primary care for low-income seniors. *J Am Geriatr Soc*. 2006;54(7):1136–41.

50. FrailSafe - Home [Internet]. Frailsafe-project.eu. [cited 2021 Jul 1]. Available: https://frailsafe-project.eu/

51. van Dijk HM, Cramm JM, Birnie E, Nieboer AP. Effects of an integrated neighborhood approach on older people's (health-related) quality of life and well-being. *BMC Res Notes*. 2016;9(1):450

52. Liotta G, Ussai S, Illario M, O'Caoimh R, Cano A, Holland C, et al. Frailty as the future core business of Public Health: Report of the activities of the A3 Action Group of the European innovation partnership on active and healthy ageing (EIP on AHA). *Int J Environ Res Public Health*. 2018;15(12):2843.

53. Healthcare Improvement Scotland. Hospital at Home Guiding principles for service development [Internet], 2020. Available: https://ihub.scot/media/6928/2020205-hospital-at-home-guiding-principles.pdf

For peer review only - http://bmjopen.bmj.com/site/about/guidelines.xhtml

54. Duda N, Fleming C, Kirwan B, Roff B, Rich E. Evaluation of the AHRQ Healthcare Horizon Scanning System [Internet], 2016. Available: https://effectivehealthcare.ahrq.gov/sites/default/files/pdf/horizon-scan_research.pdf

55. Hall RH, Lawrence PR, Lorsch JW. Organization and environment: Managing differentiation and integration. *Adm Sci Q*. 1968;13(1):180.

56. Gallego G, Bridges JF, Flynn T, Blauvelt BM. Pcn118 predicting the future impact of emerging technologies on hepatocellular carcinoma (hcc): Measuring stakeholders' preferences with best-worst scaling. *Value Health.* 2011;14(3):A176.

57. United Kingdom government office for science. The Futures Toolkit: Tools for Futures Thinking and Foresight Across UK Government [Internet], 2014. Available: https://www.gov.uk/government/publications/futures-toolkit-for-policy-makers-and-analysts

58. Sun F, Schoelles K. AHRQ HealthCare Horizon Scanning System: A Systematic Review of Methods for Health Care Technology Horizon Scanning [Internet], 2013. Available: https://effectivehealthcare.ahrq.gov/products/horizon-scan/research-2013

59. Cuhls, K. E. Horizon Scanning in Foresight – Why Horizon Scanning is only a part of the game. Futures Foresight Sci. 2020;2:e23 https://doi.org/10.1002/ffo2.23

60. Delphi Technique in Health Sciences: A Map. Niederberger, M , Spranger, J. Frontiers in Public Health 2020 doi: 10.3389/pubh.2020.00457

LEGENDS

For peer review only - http://bmjopen.bmj.com/site/about/guidelines.xhtml

Figure 1: Common stages of horizon scanning from the Euroscan Network,[24]. This figure is licensed under the Creative Commons Attribution-NonCommercial-ShareAlike 4.0 International license (CC BT-NC-SA 4.0).

Figure 2: Integrated care models. Adapted from,[27-29].

Figure 3 Horizon scanning process chart.

Figure 4 Overview over the models and interventions detailed by the records,[45-53].

Table 1 Information sources and search terms used for signal detection.

Table 2 Intervention characteristics and considerations used to filter and prioritise models and interventions,[34] [40-41].

Table 3 Scoring of models and interventions.

For peer review only

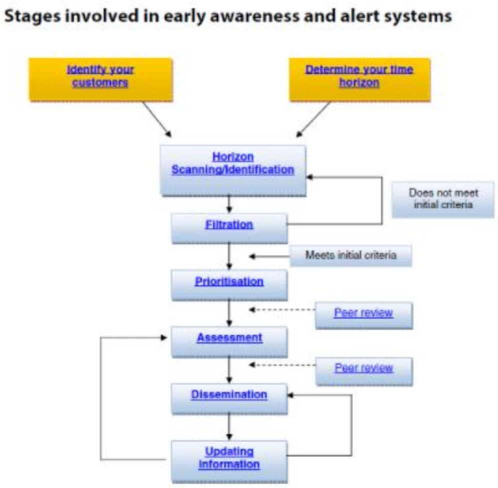

Figure 1: Common stages of horizon scanning from the Euroscan Network,[24]. This figure is licensed under the Creative Commons Attribution-NonCommercial-ShareAlike 4.0 International license (CC BT-NC-SA 4.0).

Figure 1: Common stages of horizon scanning from the Euroscan Network,[24]. This figure is licensed under the Creative Commons Attribution-NonCommercial-ShareAlike 4.0 International license (CC BT-NC-SA 4.0).

68x50mm (300 x 300 DPI)

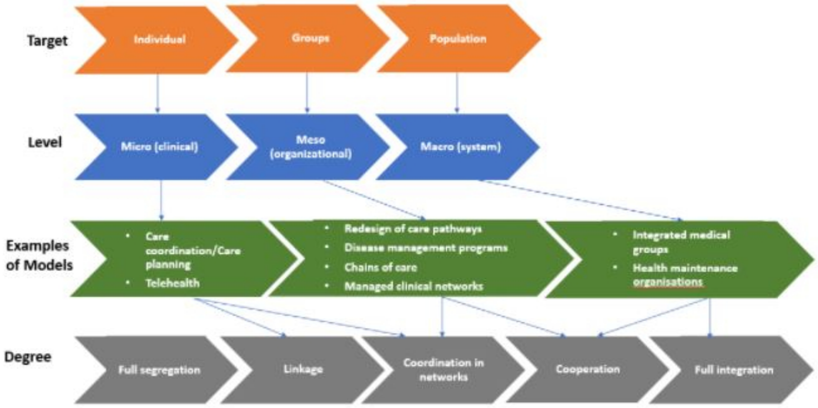

Figure 2: Integrated care models. Adapted from,[27-29]

Figure 2: Integrated care models. Adapted from,[27-29].

67x36mm (300 x 300 DPI)

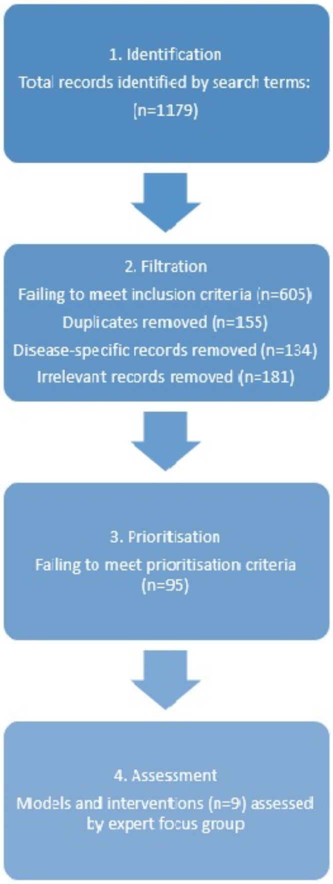

Figure 3 Horizon scanning process chart

Figure 3 Horizon scanning process chart.

66x67mm (300 x 300 DPI)

Title of ICM	PRISMA	SIPA	WICM	PACE/ON LOK	GRACE
	Program of Research to Integrate the Services for the Maintenance of Autonomy	Services intégrés pour les personnes âgées fragiles	Walcheren Integrated Care Model	Program of All-Inclusive Care for the Elderly	Geriatric Resources for Assessment and Care of Elders
Overview					
Objectives	Improve continuity and increase the efficacy and efficiency of services	Increased availability of nursing, homemakers, rehabilitation,and social work services would reduce the use and costs of institution-based services	Improve the quality and efficacy of care given to frail elderly living independently	Provide and coordinate cost-effective, comprehensive care for frail adults who want to remain in the community as long as possible but need long term assistance	Provide home-based geriatric specialised coordinated care with focus on both medical and social care (mental health)
General description	Coordination focused integrated service delivery model	Community-based system responsible for also institutional services at no additional cost. Intensive home care, 24 hour on-call availability and rapid team mobilisation	Comprehensive integrated model with focus on the family physician's role as a coordinator of care, proactive detection and assessment of needs for independently living frail elderly	Fully integrated community based model to provide all types of services at one adult day health center	Primary care service program for frail older adults especially those who have low income
Degree of integration: Linkage/Coordination in networks/Cooperation/Full integration	Coordinated provider model: Joint Governing Board for policy, service provision and resource allocation decisions (strategic). Service coordination committee for monitoring of service coordination(tactical) with multidisciplinary team of practitioners (clinical)	Fully integrated provider model: Each SIPA site, 1 program director and administrative support personnel helped to determine its own budget, implementation plan for the patients/services required, partnering agreements and deployment of human resources	Partially integrated provider model: Steering group (umbrella organinsation) consists of representatives from all organisations for the necessary provider network. Family physician is part of the network and refers the patient accordingly.	Fully integrated provider model: providing primary care, specialty care, adult day care, home care, hospital care, nursing-home care, medication oversight, and transportation to medical appointments all at an adult day health care center	Integrated provider model on community medical and social care level; co-ordinated model with acute sector and family physician
Source	(MacAdam, 2015)	(Béland et al., 2006)	(Looman et al., 2014)	(Hansen, 2008)	(Counsell et al., 2006)

Title of Intervention	EuFrailSafe	Integrated neighborhood approach(INA)	Massive Open Online Courses (MOOCs) in Frailty	Hospital at home
Overview				
Objectives	Use of advanced technology for frailty assessment, monitoring and developement of personalised frailty health plans to prevent adverse outcomes.	Improve the frail elderly and their self-management skills by connecting with informal networks, available neighbourhood resources before seeking professional help.	Empower the frail and their caregivers by informing them about the aging process in order to increase functional capacity and independence.	To provide individualised care and prevent hospital acquired infections, and functional decline due to stress from transitioning between the departments. To free up hospital beds.
General description	Smart garment (wearable sensor device to monitor medical parameters), indoor localisation application (bluetooth monitoring of movement patterns of the frail at home), games (monitor coodination, decision making skills and reflex)	INA team: Community worker examines the frail elderly needs through interviews, implements individualised care plans involving informal caregivers such as neighbours and volunteers who help to monitor the frail elderly person.	Free online courses providing material as well as a discussion platform for the frail elderly and caregivers to stay informed and be part of an online network.	Emergency access to hospital treatments/diagnostics are provided at home of the frail. Usually consultant-led and care delivered by a multidisciplinary team with the GP.
Degree of integration: Linkage/Coordination in networks/Cooperation/Full integration	Coordination in networks	Coordination in networks+ Coorperation	Coordination in networks	Full integration+ Coordination+ Cooperation
Source	(FrailSafe - Home, 2020)	(van der Heide et al., 2018)	(Liotta et al., 2018)	(Healthcare Improvement Scotland, 2020)

Figure 4 Overview of prioritised system-based integrated care models and community-based interventions,[41-49]

Figure 4 Overview over the models and interventions detailed by the records,[45-53].

84x67mm (300 x 300 DPI)

Preferred Reporting Items for Systematic reviews and Meta-Analyses extension for Scoping Reviews (PRISMA-ScR) Checklist

SECTION	ITEM	PRISMA-ScR CHECKLIST ITEM	REPORTED ON PAGE #
**TITLE**			
Title	1	Identify the report as a scoping review.	
**ABSTRACT**			
Structured summary	2	Provide a structured summary that includes (as applicable): background, objectives, eligibility criteria, sources of evidence, charting methods, results, and conclusions that relate to the review questions and objectives.	
**INTRODUCTION**			
Rationale	3	Describe the rationale for the review in the context of what is already known. Explain why the review questions/objectives lend themselves to a scoping review approach.	
Objectives	4	Provide an explicit statement of the questions and objectives being addressed with reference to their key elements (e.g., population or participants, concepts, and context) or other relevant key elements used to conceptualize the review questions and/or objectives.	
**METHODS**			
Protocol and registration	5	Indicate whether a review protocol exists; state if and where it can be accessed (e.g., a Web address); and if available, provide registration information, including the registration number.	
Eligibility criteria	6	Specify characteristics of the sources of evidence used as eligibility criteria (e.g., years considered, language, and publication status), and provide a rationale.	
Information sources*	7	Describe all information sources in the search (e.g., databases with dates of coverage and contact with authors to identify additional sources), as well as the date the most recent search was executed.	
Search	8	Present the full electronic search strategy for at least 1 database, including any limits used, such that it could be repeated.	
Selection of sources of evidence†	9	State the process for selecting sources of evidence (i.e., screening and eligibility) included in the scoping review.	
Data charting process‡	10	Describe the methods of charting data from the included sources of evidence (e.g., calibrated forms or forms that have been tested by the team before their use, and whether data charting was done independently or in duplicate) and any processes for obtaining and confirming data from investigators.	
Data items	11	List and define all variables for which data were sought and any assumptions and simplifications made.	
Critical appraisal of individual sources of evidence§	12	If done, provide a rationale for conducting a critical appraisal of included sources of evidence; describe the methods used and how this information was used in any data synthesis (if appropriate).	
Synthesis of results	13	Describe the methods of handling and summarizing the data that were charted.	

For peer review only

St. Michael's
Inspired Care.
Inspiring Science.

For peer review only - http://bmjopen.bmj.com/site/about/guidelines.xhtml

SECTION	ITEM	PRISMA-ScR CHECKLIST ITEM	REPORTED ON PAGE #
**RESULTS**			
Selection of sources of evidence	14	Give numbers of sources of evidence screened, assessed for eligibility, and included in the review, with reasons for exclusions at each stage, ideally using a flow diagram.	
Characteristics of sources of evidence	15	For each source of evidence, present characteristics for which data were charted and provide the citations.	
Critical appraisal within sources of evidence	16	If done, present data on critical appraisal of included sources of evidence (see item 12).	
Results of individual sources of evidence	17	For each included source of evidence, present the relevant data that were charted that relate to the review questions and objectives.	
Synthesis of results	18	Summarize and/or present the charting results as they relate to the review questions and objectives.	
**DISCUSSION**			
Summary of evidence	19	Summarize the main results (including an overview of concepts, themes, and types of evidence available), link to the review questions and objectives, and consider the relevance to key groups.	
Limitations	20	Discuss the limitations of the scoping review process.	
Conclusions	21	Provide a general interpretation of the results with respect to the review questions and objectives, as well as potential implications and/or next steps.	
**FUNDING**			
Funding	22	Describe sources of funding for the included sources of evidence, as well as sources of funding for the scoping review. Describe the role of the funders of the scoping review.	

JBI = Joanna Briggs Institute; PRISMA-ScR = Preferred Reporting Items for Systematic reviews and Meta-Analyses extension for Scoping Reviews.

* Where *sources of evidence* (see second footnote) are compiled from, such as bibliographic databases, social media platforms, and Web sites.

† A more inclusive/heterogeneous term used to account for the different types of evidence or data sources (e.g., quantitative and/or qualitative research, expert opinion, and policy documents) that may be eligible in a scoping review as opposed to only studies. This is not to be confused with *information sources* (see first footnote).

‡ The frameworks by Arksey and O'Malley (6) and Levac and colleagues (7) and the JBI guidance (4, 5) refer to the process of data extraction in a scoping review as data charting.

§ The process of systematically examining research evidence to assess its validity, results, and relevance before using it to inform a decision. This term is used for items 12 and 19 instead of "risk of bias" (which is more applicable to systematic reviews of interventions) to include and acknowledge the various sources of evidence that may be used in a scoping review (e.g., quantitative and/or qualitative research, expert opinion, and policy document).

From: Tricco AC, Lillie E, Zarin W, O'Brien KK, Colquhoun H, Levac D, et al. PRISMA Extension for Scoping Reviews (PRISMAScR): Checklist and Explanation. Ann Intern Med. 2018;169:467–473. doi: 10.7326/M18-0850.

For peer review only - http://bmjopen.bmj.com/site/about/guidelines.xhtml

COREQ (COnsolidated criteria for REporting Qualitative research) Checklist

A checklist of items that should be included in reports of qualitative research. You must report the page number in your manuscript where you consider each of the items listed in this checklist. If you have not included this information, either revise your manuscript accordingly before submitting or note N/A.

Topic	Item No.	Guide Questions/Description	Reported on Page No.
**Domain 1: Research team and reflexivity**			
Personal characteristics			
Interviewer/facilitator	1	Which author/s conducted the interview or focus group?	
Credentials	2	What were the researcher's credentials? E.g. PhD, MD	
Occupation	3	What was their occupation at the time of the study?	
Gender	4	Was the researcher male or female?	
Experience and training	5	What experience or training did the researcher have?	
Relationship with participants			
Relationship established	6	Was a relationship established prior to study commencement?	
Participant knowledge of the interviewer	7	What did the participants know about the researcher? e.g. personal goals, reasons for doing the research	
Interviewer characteristics	8	What characteristics were reported about the inter viewer/facilitator? e.g. Bias, assumptions, reasons and interests in the research topic	
**Domain 2: Study design**			
Theoretical framework			
Methodological orientation and Theory	9	What methodological orientation was stated to underpin the study? e.g. grounded theory, discourse analysis, ethnography, phenomenology, content analysis	
Participant selection			
Sampling	10	How were participants selected? e.g. purposive, convenience, consecutive, snowball	
Method of approach	11	How were participants approached? e.g. face-to-face, telephone, mail, email	
Sample size	12	How many participants were in the study?	
Non-participation	13	How many people refused to participate or dropped out? Reasons?	
Setting			
Setting of data collection	14	Where was the data collected? e.g. home, clinic, workplace	
Presence of non-participants	15	Was anyone else present besides the participants and researchers?	
Description of sample	16	What are the important characteristics of the sample? e.g. demographic data, date	
Data collection			
Interview guide	17	Were questions, prompts, guides provided by the authors? Was it pilot tested?	
Repeat interviews	18	Were repeat inter views carried out? If yes, how many?	
Audio/visual recording	19	Did the research use audio or visual recording to collect the data?	
Field notes	20	Were field notes made during and/or after the inter view or focus group?	
Duration	21	What was the duration of the inter views or focus group?	
Data saturation	22	Was data saturation discussed?	
Transcripts returned	23	Were transcripts returned to participants for comment and/or	

For peer review only

Topic	Item No.	Guide Questions/Description	Reported on Page No.
		correction?	
**Domain 3: analysis and findings**			
Data analysis			
Number of data coders	24	How many data coders coded the data?	
Description of the coding tree	25	Did authors provide a description of the coding tree?	
Derivation of themes	26	Were themes identified in advance or derived from the data?	
Software	27	What software, if applicable, was used to manage the data?	
Participant checking	28	Did participants provide feedback on the findings?	
Reporting			
Quotations presented	29	Were participant quotations presented to illustrate the themes/findings? Was each quotation identified? e.g. participant number	
Data and findings consistent	30	Was there consistency between the data presented and the findings?	
Clarity of major themes	31	Were major themes clearly presented in the findings?	
Clarity of minor themes	32	Is there a description of diverse cases or discussion of minor themes?	

Developed from: Tong A, Sainsbury P, Craig J. Consolidated criteria for reporting qualitative research (COREQ): a 32-item checklist for interviews and focus groups. *International Journal for Quality in Health Care*. 2007. Volume 19, Number 6: pp. 349 – 357

Once you have completed this checklist, please save a copy and upload it as part of your submission. DO NOT include this checklist as part of the main manuscript document. It must be uploaded as a separate file.

For peer review only

Supplemental material

Supplementary table 1: Backgrounds of participants

**Regional health authority**	**N**	**Background of participant**
Central Norway	4	Senior researcher/ Nurse/ Professor<br>Physiotherapist/ Project manager<br>Advisor<br>Senior Advisor/ Phd-fellow/ Nurse
West	1	Research coordinator/ Nurse specialist (oncology)
South-East	2	Specialist in internal medicine and geriatrics/ Chief physician/<br>Professor<br>Leader of community services development/ Nurse
Total	7	

For peer review only

Supplementary figure 1: Interview guide

"Horizon scanning of healthcare delivery models within services targeted to the frail elderly"

Focus group agenda

Overall aim of this study

To evaluate if horizon scanning can be used to help decision makers to fill in the knowledge gaps, address issues such as system fragmentation, as well as contribute to innovation of the healthcare delivery services targeted at the frail elderly.

Program

No.	Topic	Focus	Time
1.	Introduction	- Brief description of the study's purpose<br>- Participants' presentation of themselves	1530-1540
2.	Horizon scanning process	- Description of horizon scanning steps<br>- Presentation of results from the scan	1540-1550
3.	System-based models	- Introduction of each model<br>- Discussion<br>- Evaluation	1550-1630
4.	Community-based interventions	- Introduction of each intervention<br>- Discussion<br>- Evaluation	1630-1650
5.	Horizon scanning methodology	- Discussion and evaluation	1650-1700

Interview guide

Do you have any potential conflicts of interest, such as ongoing research or other intellectual / financial interests with organizations related to the models/interventions discussed in this interview?

Yes No

If yes, please describe:

List of innovations

To avoid miscommunication we define new innovations as a possible new way of organizing services, a new mechanism in the service process, changes in the system that increase access to more comprehensive services for frail elderly as well as a new application of existing intervention (s), or other current innovations.

The list is structured after system-based and community-based with an aim to create a better overview for discussion and evaluation. The division is not unambiguous as the integrated care models and community-based interventions do contain overlapping elements.

For peer review only

The innovations placed under "system-based" contain core traits of integrated care models specific for frail elderly on a system/population large scale level.

The innovations placed under "community-based" contain traits that allow for the frail to live independently in the community. These have a "door in" approach and are on a more local/community small scale level. This does not mean the community-based are not involved in system level decision making and vice versa.

Discussion & Evaluation on list of innovations

Please reply if you are aware of the mentioned innovations, and, if applicable, leave a comment on the various innovations.

System-based Innovation	Do you know this?	Additional comments
PRISMA	Yes/No	
SIPA	Yes/No	
WICM	Yes/No	
PACE	Yes/No	
GRACE	Yes/No	

Community-based Innovation	Do you know this?	Additional comments
EuFrailSafe	Yes/No	
INA	Yes/No	
MOOCs	Yes/No	
Hospital at Home	Yes/No	

Based on the description and your experience, please rate them on a scale of *low, moderate, and high* accordingly to

i) Level of innovation: degree of novelty, filtration of services from that of common practice.

ii) Probability that the innovation will be further implemented in the next 2-10 years: to see which innovations most likely to be in the horizon of integrated healthcare services for frail elderly. *Things to consider here are resource implications, expected utilisation and availability of the innovation across different geographical areas, actions required before implementation can take place, time, and investment in training of personnel, cooperation of stakeholders and ethical and accessibility issues.*

iii) Likely impact on frail elderly: importance/quality of the innovation. *Things to consider here are the innovation's ability to solve current service issues such as disease-focused treatments, long waiting times, poor exchange of knowledge/collaboration among health workers as a result of not having a shared electronic health record, insufficient staff numbers, lack of guidelines and accountability for care management, absence of professional expertise regarding the patient's health condition, lack of clarity with regards to health personnel's duties and responsibilities as well as a failure in offering updates to patients and their families, along with preparing them for future care transfers.*

For peer review only

System-based Innovation	Level of innovation	Comments	Probability of implementation in the next 2-10 years	Comments	Likely impact on frail elderly	Comments
PRISMA						
SIPA						
WICM						
PACE						
GRACE						

Community-based Innovation	Level of innovation	Comments	Probability of implementation in the next 2-10 years	Comments	Likely impact on frail elderly	Comments
EuFrailSafe						
INA						
MOOCs						
Hospital at Home						

Discussion & Evaluation on horizon scanning methodology

What are the current methods you use for making decisions in healthcare service delivery?

What do you think of horizon scanning as a tool for decision making in healthcare service delivery?

What would be the possible strengths and weaknesses of using such a tool?

Any further comments?

For peer review only

Supplementary table 2: Illustrative quotes from Qualitative assessment

**Shifting away from specialist acute reactive care**	**EuFrailSafe**<br>"It is a trend and a need to focus on prevention with the use of technology" Informant 5	**Hospital at Home**<br>"If it is well organised within the municipality and we are familiar with the patient's background and medical issues, then it is best and definitely possible to treat them at home" Informant 4	**MOOCs**<br>"This seems to be innovative as it is prevention focused and more customised for frail people and their caregivers, plus the information is easily accessible" Informant 3	**WICM**<br>"I think it would be beneficial if the frail elderly patients were screened early at the doctor's office to avoid hospital admissions" Informant 6
**Silos**	**PRISMA & SIPA**<br>"I like that there is a defined team responsible for the patient's cate and the focus is on coordination" Informant 3	**INA**<br>"A social worker who acts as a coordinator and does assessments at the frail person's home while involving neighbours and volunteers is new and innovative. I have never heard about it" Informant 7		
**Service gaps and duplications**	**EuFrailSafe**<br>"The virtual platform and use if monitoring devices allow for better clinical follow-up and care" Informant 6	**PRISMA & SIPA**<br>"The connection between the specialist service and the primary health service is poor, we do not have any communication while in these models, there is a team and a platform they use to meet and plan and the care for the patient. I think it is a great idea especially for the frail with complex health problems" Informant 7	**GRACE & PACE**<br>"These models seem to have good collaboration routines between the specialist and the primary health services as well as interdisciplinary teams within the primary health service which I feel is important" Informant 3	
**Competence requirements**	**MOOCs**<br>"I think it would be useful as you get knowledge about the health condition you are struggling with and support from others" Informant 5	**WICM**<br>"The idea of a nurse practitioner and family physician teaming up to do geriatric assessments for frailty and early deterioration among elderly is innovative" Informant 1		
**Greater patient and network involvement**	**WICM**<br>"There is active caregiver support and involvement, that is innovative" Informant 1	**GRACE & PACE**<br>"I believe that in the future with the lack of healthcare personnel and a growing number of elderlies, initiatives that involve the	**INA**<br>"It would be useful and something we would need in the future as there is a focus on strengthening social networks in a local	

For peer review only

		network surrounding the frail patient will become essential" Informant 5	environment, where a neighbourhood takes responsibility for the care of the frail" Informant 2	

For peer review only
