# Supplementary material for: Models of integrated care for older people with frailty: a horizon scanning review
Source: BMJ Open. 2022 Apr 8;12(4):e060142. doi: 10.1136/bmjopen-2021-060142 (PMC8996021; doi:10.1136/bmjopen-2021-060142)
Supplement: Reviewer comments [file bmjopen-2021-060142.reviewer_comments.pdf]

PEER REVIEW HISTORY

BMJ Open publishes all reviews undertaken for accepted manuscripts. Reviewers are asked to complete a checklist review form (**http://bmjopen.bmj.com/site/about/resources/checklist.pdf**) and are provided with free text boxes to elaborate on their assessment. These free text comments are reproduced below.

ARTICLE DETAILS

**TITLE (PROVISIONAL)**	Models of integrated care for older people with frailty: A horizon scanning review
**AUTHORS**	A Kjelsnes, Ashwanee; Feiring, Eli

VERSION 1 – REVIEW

**REVIEWER**	Philip Hines<br>European Medicines Agency, SciRS
**REVIEW RETURNED**	05-Aug-2021

**GENERAL COMMENTS**	Dear authors,<br><br>Well done on your clearly written and concise description of your research. It was a comparative pleasure to peer review. I reviewed it mainly for its methodology rather than results in integrated frailty as I have no expertise here.<br><br>My overreaching comment is that this is slightly more a literature review of existing integrated care methods than horizon scanning for new methods. However you acknowledge this as a limitation and rightly highlight the difficulty of scanning for healthcare approaches which are, by definition, established rather than new tech or trends.<br><br>Limitations: There were seven participants given two hours to deliberate and score. This limits the validity of the results through increased bias, as you rightly recognise the the text. In an ideal world a Delphi-like approach could have been taken, with a first anonymous review, scoring and commenting, before the two hour discussion.<br><br>Here are some more specific comments:<br><br>- Consider changing "The first step includes mapping signals of innovation with the use of literature reviews, including then disseminated and evaluated,[26]. reviews of grey lit..." To<br>"The first step often includes mapping signals of innovation with the use of literature reviews, including then disseminated and evaluated,[26]. reviews of grey lit<br><br>- Consider changing :" The state is responsible for specialist health care, which is administrated by four Regional Health Authorities (RHA). The lack of communication between the two tiers of gove..." to "The central state is responsible for secondary and specialist health care, which is administrated by four Regional Health Authorities (RHA). The lack of communication between the two tiers of gove

	- In methods, rather than just in the supp. Table 2, please specify what databases were searched, the search terms, a table of the inclusion and exclusion criteria, and the date range. This should be as far as possible according to the PRISMA guideline (although i recognise that this was not a systematic literature review): https://doi.org/10.1186/s13643-020-01542-z<br><br>- Consider amending the sentence to include integrated care and frailty as otherwise, on this criteria alone, it seems you could have included multiple irrelevant publications "Community-based interventions. Local or community level-based interventions; Living-at-home; Promote indepen...'<br><br>- Regarding the section 'Quantitative scores' please clarify how you scored I.e. whether you used an average score approach from the seven participants.<br><br><br>- Supplementary table 3<br>Are the following all required for prioritisation, or can a subset only be met? "System level integrated care models: Are the following aspects included: a centralized point of entry, geriatric evaluation, case management, multidisciplinary teams, multidisciplinary guidelines and meetings, digitalized patient files, network framework? ,[36] "<br><br>- Regarding figure 4<br>It is unclear what information about the selected models was provided to the participants in advance. Please clarify that they recieved the full publications

**REVIEWER**	Anne Hendry<br>NHS Lanarkshire, R&D
**REVIEW RETURNED**	27-Aug-2021

**GENERAL COMMENTS**	Thank you for the opportunity to review this paper. I am afraid I have several reservations and am unable to recommend publication.<br><br>The paper presents outputs of a literature review on models of integrated care for frailty conducted to inform the horizon scanning process. There are insufficient details to assess the rigour of the review. I note the references do not acknowledge the European Joint Action on Frailty and the related scientific outputs that are a result of horizon scanning by experts from 27 countries from 2017-2019.<br><br>The nine selected examples mix comprehensive models of integrated chronic care with examples of more discrete interventions that provide specific components of integrated care such as education, ICT enabled support for self management or acute care at home.<br><br>The 7 participants in the focus group offer a rather narrow professional perspective and there is no policy, economic, patient or public involvement in assessment and prioritisation of the examples.<br><br>There is a single focus group of 2 hours duration to discuss and prioritise these complex interventions / approaches and also explore the utility of the horizon scanning methodology. This seems rather ambitious as noted by participants.

	Assertions about the need for legislation to enable implementation are not well evidenced.<br><br>The references to frail elderly should be reframed as older people with frailty.

VERSION 1 – AUTHOR RESPONSE

Reviewer: 1

Mr. Philip Hines, European Medicines Agency

Comments to the Author:

Dear authors,

Well done on your clearly written and concise description of your research. It was a comparative pleasure to peer review. I reviewed it mainly for its methodology rather than results in integrated frailty as I have no expertise here.

My overreaching comment is that this is slightly more a literature review of existing integrated care methods than horizon scanning for new methods. However you acknowledge this as a limitation and rightly highlight the difficulty of scanning for healthcare approaches which are, by definition, established rather than new tech or trends.

Limitations: There were seven participants given two hours to deliberate and score. This limits the validity of the results through increased bias, as you rightly recognise the the text. In an ideal world a Delphi-like approach could have been taken, with a first anonymous review, scoring and commenting, before the two hour discussion.

First, thanks a lot for very helpful and constructive comments!

We fully agree to this point:

- **We have provided a better description of our approach in the Methods section. The participants were informed about the different models by e-mail prior to the discussion session and asked to score and evaluate the different models independently, but we did not collect their evaluations. The approach was a pragmatic choice given the time- and resource limits of this pilot horizon scanning project. In addition, as we were aware of the time and resource limits of this project, we tried to ensure that each participant had various roles and could represent various work settings (For example, one of the participants was a nurse but also a senior researcher and a professor and thus she was able to share insights from a**

clinical and academia point of view). These points are now made explicit in the Methods section, “Focus group: Assessment of records “/ Results section, “Evaluation”.

- **In the Limitation section, we have commented on the ideal design and the use of a Delphi-like approach contrasted to our approach, and we have pointed out how the use of a Delphi technique may strengthen validity of findings.**

Here are some more specific comments:

- Consider changing "The first step includes mapping signals of innovation with the use of literature reviews, including then disseminated and evaluated,[26]. reviews of grey lit..." To

"The first step often includes mapping signals of innovation with the use of literature reviews, including then disseminated and evaluated,[26]. reviews of grey lit

We have done this.

- Consider changing :" The state is responsible for specialist health care, which is administrated by four Regional Health Authorities (RHA). The lack of communication between the two tiers of gove..." to "The central state is responsible for secondary and specialist health care, which is administrated by four Regional Health Authorities (RHA). The lack of communication between the two tiers of gove

We have done this.

- In methods, rather than just in the supp. Table 2, please specify what databases were searched, the search terms, a table of the inclusion and exclusion criteria, and the date range. This should be as far as possible according to the PRISMA guideline (although i recognise that this was not a systematic literature review): https://doi.org/10.1186/s13643-020-01542-z

We have now provided this information in the main text in the section “Search strategy”. We have provided a COREQ guideline form and a PRISMA form as supplementary files.

- Consider amending the sentence to include integrated care and frailty as otherwise, on this criteria alone, it seems you could have included multiple irrelevant publications "Community-based interventions. Local or community level-based interventions; Living-at-home; Promote indepen...'

We have done this.

- Regarding the section 'Quantitative scores' please clarify how you scored I.e. whether you used an average score approach from the seven participants.

We have clarified the scoring in the section “Quantitative scores”.

- Supplementary table 3

Are the following all required for prioritisation, or can a subset only be met? "System level integrated care models: Are the following aspects included: a centralized point of entry, geriatric evaluation, case management, multidisciplinary teams, multidisciplinary guidelines and meetings, digitalized patient files, network framework? ,[36] "

We have clarified how we did the prioritisation in the sections Inclusion and exclusion criteria and Prioritisation – we used all criteria to evaluate the papers in the first round of prioritisation. We found that only a sub-set of the criteria was met by the different models and we prioritised those models that had the highest scored to be discussed in the focus group.

- Regarding figure 4

It is unclear what information about the selected models was provided to the participants in advance. Please clarify that they recieved the full publications

We have clarified this point in the section Focus groups: Assessment of records, Data collection. The participants got a summary of the different models as well as the full reference to the publications. At the start of the focus group session, the summary of the different models was presented and the participants were asked if they had any questions. This was done to clear up any misunderstandings or questions they had about the models.

Reviewer: 2

Dr. Anne Hendry, NHS Lanarkshire, University of the West of Scotland

Comments to the Author:

Thank you for the opportunity to review this paper. I am afraid I have several reservations and am unable to recommend publication.

The paper presents outputs of a literature review on models of integrated care for frailty conducted to inform the horizon scanning process. There are insufficient details to assess the rigour of the review. I note the references do not acknowledge the European Joint Action on Frailty and the related scientific outputs that are a result of horizon scanning by experts from 27 countries from 2017-2019.

Thank you for your comments!

We have now provided information about the literature search phase of the horizon scanning in the Methods section (rather than just in the Supplementary files). Moreover, we have provided a PRISMA form as a Supplementary file.

We have also acknowledged the European Joint Action on Frailty (ADVANTAGE JA) outputs in the Introduction and pointed out how the recent systematic review (Hendry et al 2019) concludes: "The literature review identified few models of integrated care specifically designed to prevent and tackle frailty in the community and at the interface between primary care and secondary care".

We have also noticed that according to this paper, "Further research is required to understand how to scale up integrated care for frailty in different systems…" .

Our present study is a pilot horizon scan and is conducted in a given national healthcare system, the Norwegian NHS. The aim of our study is different from the systematic review given by the ADFVANTAGE JA: We wanted to know if we were able to identify innovative models not yet implemented or tested in a Norwegian setting with a horizon scanning methodology. We did not search for articles that explicitly assessed the impact of models; rather we searched for emerging models that we considered to have the potential to address system fragmentation issues in a Norwegian setting. We filtered the findings before prioritising models and interventions. We used a set of prioritisation criteria - resulting from previous empirical studies that have suggested key components of an effective model of integrated care for frailty - to choose models that subsequently were discussed by expert healthcare professionals in a focus group. Many of the models reported in Hendry et al systematic literature review were prioritised to be discussed by the focus group in our study, on the basis of the literature review phase of the pilot horizon scan. Although these models were developed a while ago, implemented and tested for efficiency in another context, we considered the models to be sufficiently innovative and new to the Norwegian context to be included. This is further confirmed by the participants who despite working closely with frail elderly, were unaware of the models and interventions discussed during the focus group session. The participants regarded the models and interventions as "new" and "innovative".

Thus, we did not aim to do a systematic literature review (as noticed by Reviewer 1). Rather, we aimed to explore the use of horizon scanning methodology to scan for innovative approaches (not yet implemented and outcome tested) to managing frailty in a Norwegian setting. We have stated our study aim more clearly in the new version of the paper to avoid further misunderstandings.

The nine selected examples mix comprehensive models of integrated chronic care with examples of more discrete interventions that provide specific components of integrated care such as education, ICT enabled support for self management or acute care at home.

In the paper, we separated between system level models (n=5) and community level interventions (n=4). We have now clarified how the different models and interventions differ and how the models and interventions are tailored to key points in the frailty care pathway.

The 7 participants in the focus group offer a rather narrow professional perspective and there is no policy, economic, patient or public involvement in assessment and prioritisation of the examples.

We have commented on this limitation and we have provided more reflection on this point in the Limitation section. We report from a horizon scanning pilot that was conducted within strict resource limits. In an ideal setting, all stakeholders should be represented and a Delphi approach might be used to collect judgements on a broad scale and even to identify consensus/disagreements. However, the participants had diverse backgrounds and thus the models and interventions presented during the focus group session were evaluated from various standpoints. The standpoints included clinical, political, academia/research. We have commented on this point in the present paper.

There is a single focus group of 2 hours duration to discuss and prioritise these complex interventions / approaches and also explore the utility of the horizon scanning methodology. This seems rather ambitious as noted by participants.

We totally agree and we have commented further on this point in the Limitation section.

Assertions about the need for legislation to enable implementation are not well evidenced.

We have elaborated more on this point in the Discussion section. Further, we have provided more information about the Norwegian NHS in the Setting section. In Norway, specialist care is the responsibility of the state, while autonomous municipalities are responsible for the provision and financing of primary care. Although a Coordination Reform (2012) established mandatory network governance to improve coordination between specialist and primary care, integrated care involving different levels is hindered by lack of formalised coordination and cooperation between hospitals and municipalities, according to the Norwegian Health and Hospital Plan 2020-2023. We have substantiated this point in the paper.

The references to frail elderly should be reframed as older people with frailty.

We have done this.
